# Design of S/C/X/Ku frequency band five notch broadband filter based on novel coupled signal path

**DOI:** 10.1371/journal.pone.0315007

**Published:** 2025-03-17

**Authors:** Guojin Li, Wenxian Yang, Jingchang Nan

**Affiliations:** College of Electronics and Information Engineering, Liaoning Technical University, Huludao, Liaoning, China; Griffith University - GC Campus: Griffith University - Gold CoastCampus, AUSTRALIA

## Abstract

This article present the design of a broadband filter structure spanning the S, C, X, and Ku uplink bands based on a novel coupling signal path generation. The core design features a centrally symmetric serial via hole array, microstrip line, and curved coplanar waveguide. This structure offers advantages such as low loss band pass, low frequency stopband in the S band, and stable transmission zeros while providing a broadband passband. Additionally, to address the signal suppression requirements in commercial communication frequencies, such as 5G base stations, 6G IMT, satellite TV, and Starlink, across the S, C, X, and Ku uplink bands, a dual-band notch structure for the C band and three improved signal suppression structures are proposed. Finally, the design achieves miniaturization and integration while ensuring excellent performance across all five independent stopbands. Experimental results closely match simulation findings: the filter exhibits a –3 dB bandwidth of 2.4–14.3 GHz with a relative bandwidth of up to 141*%*. Each of the five suppression depths reaches –15 dB, occupying a compact footprint of only 14×10.8mm^2^,with a relative wave guide area of 0.38 × 0.29λ^2^.

## Introduction

In civilian communications, the current trend in wireless RF systems is towards integrating multiple frequency bands as compactly as possible. However, the challenge of coping with increasing interference from narrowband commercial signals [1–3] persists due to the extremely wide operating bandwidth. In recent years, rapid developments in commercial communications within the S/C/X/KU band have included the widespread adoption of 5G base station and user frequency bands following the maturity of 5G technology, as well as frequency bands designated for the 6G mobile communication IMT system from 2023. Additionally, the commercial communication signals in the X and Ku bands for user uplink include two key frequencies: the 12.75–13.25 GHz band used by Tesla to support the Starlink project [2], and Fixed Wireless Systems (FWS) used in satellite television communications [3]. With the increasing demand for 5G smart devices and base stations, other wireless system [4], particularly those with broadband characteristics, are experiencing heightened interference from 5G wireless communications. To meet the requirements of wideband systems in suppressing interference from commercial communications, this paper aims to design a miniaturized bandstop/bandpass filter for the S/C/X/KU band (2–15 GHz). Its passband covers S/C/X bands and parts of the Ku uplink band used by users in the frequency range of 12–15 GHz, while suppressing commercial communication frequency bands including 5G base station services (3.7–4 GHz), 5G WiFi U-NII-2 protocol (5.25–5.725 GHz), 6G IMT systems (6.425–7.125 GHz) [5], satellite communication FWS systems (10.7–11.7 GHz), and Starlink user uplink signals (12.75–13.25 GHz).

The SVA-ML-CPW structure proposed in this manuscript enables the realization of a dual-signal path filter with five highly robust passband modes and two transmission zeros. The proposed Hexa-band filter has six passbands and five notch bands simultaneously. Moreover, by incorporating a dual paper clip structure and multiple enhanced notch structures proposed in this manuscript, a five-notch broadband bandpass filter is achieved, all while maintaining low passband loss, precise notch selection, and excellent stability. The proposed microstrip bandpass filter is highly suitable for RF transmission/reception systems that require the exclusion of commercial interference signals and the collection of broadband signals. The filter features a compact size and easy integration. It is ideal for precise information extraction in complex signal environments. For example, in hospitals, it can reject mobile phone signals during internal wireless system communications, facilitating accurate patient positioning and precise transmission of patient information. Additionally, in the emerging urban delivery drone systems, this filter can reduce interference from 5G/6G and some satellite signals, ensuring that the drones only receive control signals. The filter also has other potential applications, such as high-user-density indoor information collection and ground-based phased array receiver noise reduction.

## Related work

Compact structure, small size, wide bandwidth, low loss, and spurious response suppression are critical performance parameters for the design and development of parallel-plate wideband filters [6–9]. Broadband filters typically employ three main design approaches. The first involves cascading high-pass and low-pass filters [10], which, while straightforward in design, may result in an overall size that is too large. Another common approach utilizes traditional multimode resonators with overlapping modes; however, cross-coupling [11] requires larger feeder dimensions and precise manufacturing to ensure stable passbands. Alternatively, using open-circuit stubs and cascaded impedance step branches [12,13] as another type of multimode resonator achieves low insertion loss but demands very long lengths of impedance step branches for cascading. Moreover, its asymmetric structure complicates mathematical analysis when incorporating subsequent spurious response structures.

The third design approach utilizes a multi-mode resonator structure formed by microstrip line-coplanar waveguide (ML-CPW), where the vertical coupling between the top and bottom layers significantly reduces its size. Such resonators are commonly used for achieving broadband characteristics [14,15]. Similarly, vertical coupling using continuous circular and defected ground structure (DGS) can also achieve broadband characteristics, but compared to the ML-CPW structure, it results in higher passband insertion loss [16–18]. In contrast to traditional Butterworth-type filters, these multi-mode resonators exhibit an elliptic-like response [19], resulting in better stopband attenuation. However, their drawback lies in typically being driven by only three modes. Even the recently proposed five mode filters are based on the stepped impedance structure of ML-CPW, generating three modes and utilizing the dispersion effect to produce five higher-order harmonics. This leads to insufficient strength in the high-frequency passband modes, resulting in a trade-off between minimum insertion loss and bandwidth. In contrast, the bandpass filter proposed in this paper, utilizing the SVA-ML-CPW structure, achieves five independent and highly robust high-frequency passband modes through two signal paths. Moreover, in recent years, notch filters typically feature no more than three notches, as adding more notch structures increases the filter’s insertion loss, limiting the number of notches to a maximum of four. However, due to the low-loss passband of the SVA-ML-CPW bandpass filter proposed in this paper, it is possible to incorporate five different notch structures, thereby achieving a five-notch wideband bandpass filter.

## Organization

This paper proposes a miniaturized ultra-wideband bandpass filter based on a novel coupled signal path. Sect 1 outlines the design process of the bandpass filter. Sect 1.1 introduces the innovative Serial via hole array-microstrip line-arc-shaped coplanar waveguide (SVA-ML-CPW) structure and discusses its necessity and related analysis. Sect 1.2 designs four additional passband modes through the ML-CPW structure. Sect 1.3 designed a reasonable five mode filter through weak coupling, providing theoretical predictions and simulation results for the frequency centers of each passband mode, verifying the effectiveness and performance advantages of the design. Sect 2 introduces the principles and design schemes of various notch filter structures, and assigns five notch structure positions to reduce mutual influence and achieve the five notch filter. Sect 2.1 designs the first notch filter to suppress 5G base station services using transmission zeros. Sect 2.2 proposes a dual-band notched dumbbell structure to achieve the second and third notch filters for filtering out 5G WiFi signals and 6G IMT system signals, respectively. Sect 2.3 introduces an open-circuit resonant ring structure for the fourth notch filter to suppress satellite television communication to ground, and a reverse S-type notch filter structure for the fifth notch filter to suppress user upload signals from the Starlink project, finally using a series complementary resonant ring to enhance selectivity. Section “Testing and verification” integrates and optimizes these structures to fabricate and test the five notch filter. Compared with other filters from recent years, this novel filter simultaneously possesses wider passband width, more notch filters, and acceptable low-loss passbands, achieving excellent notch filter performance on a highly integrated basis.

**Fig 1:**
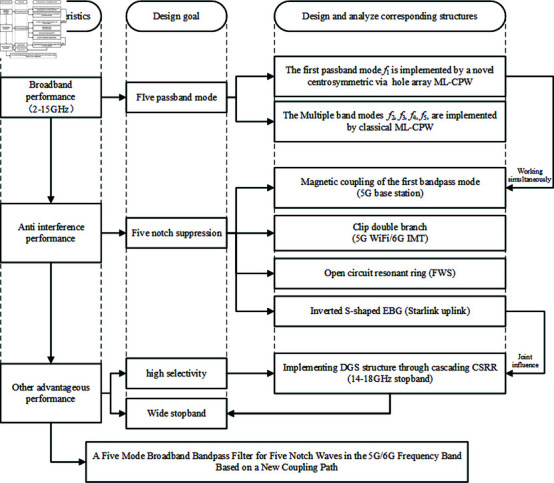
Design flow chart.

The filter design process in this paper is illustrated in Fig 1, providing characteristics, design objectives, and corresponding structures of the filter.

## 1 Novel coupling signal path design

**Fig 2:**
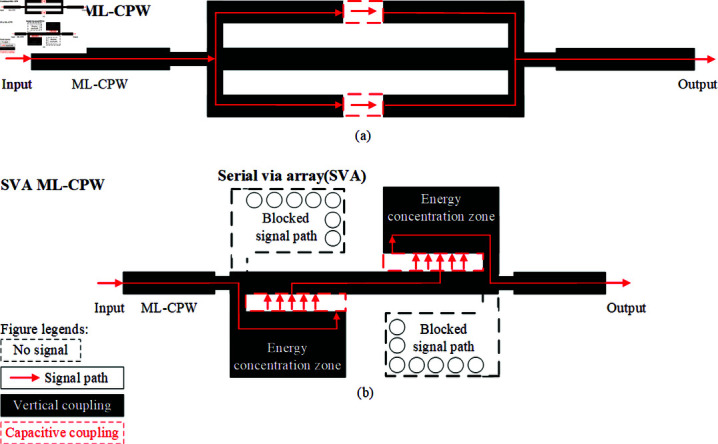
Topology of the Coupling Paths. (a) traditional ML-CPW. (b) SVA-ML-CPW.

To achieve a larger bandwidth in a smaller footprint for microstrip bandpass filters, this manuscript employs a novel coupled signal path approach. This method ensures minimal loss while effectively reducing the filter’s dimensions. Fig 2 illustrates two coupling signal paths: Fig 2(a) traditional ML-CPW and Fig 2(b) SVA-ML-CPW. The black area denotes potential vertical coupling, while the red dotted area signifies capacitive coupling. The red coupling signal path represents the longest achievable signal path for the structure, indicating that lowest passband mode beyond the maximum electrical length cannot be achieved. Typically, the traditional ML-CPW structure achieves only three passband modes, or it utilizes dispersion effects, albeit sacrificing passband insertion loss, to achieve five passband modes based on the three passband modes. To maintain low insertion loss characteristics and achieve a lower passband mode, the entire ML-CPW structure must be enlarged. This paper proposes a new coupling signal path with five passband modes implemented using a serial via hole array(SVA) in Fig 2(b), aiming to enhance functionality while miniaturizing the device. Fig 2(b) demonstrates that employing SVA to obstruct specific segments of the signal pathway and bolster coupling efficacy within the energy concentration region can facilitate an extended electrical length while minimizing the manufacturing footprint. Consequently, this approach achieves a lower Passband mode compared to traditional ML-CPW structure.

**Fig 3:**
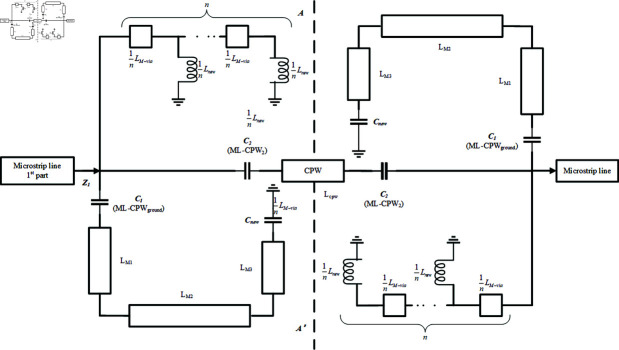
Simplified equivalent circuit diagram of SVA-ML-CPW. (a) Odd mode. (b) Even mode.

In Fig 2(b), the blocked signal path is implemented by multiple series-connected metal via arrays to ground, represented as inductance *L_n_ew*. Simultaneously, the realization of the energy concentration region in the Fig 2(b) is achieved by adjusting the capacitive coupling between microstrip lines in Fig 2(a) to a stronger vertical capacitive coupling in Fig 2(b), denoted as capacitance *Cnew*. To effectively analyze the characteristics of the new coupling signal path concerning the passband center frequency and other parameters, an equivalent circuit diagram (Fig 3) based on the novel coupling signal path is constructed, integrating the aforementioned discussion.

In Fig 3, *C*1 represents the vertical coupling between microstrip lines and the ground plane of CPW, while C2 denotes the vertical coupling between microstrip lines and CPW. ZM1 indicates the input impedance of the microstrip line. The total inductance introduced by the serial via array (SVA) is denoted as , with each individual via hole contributing an inductance of Due to the presence of inductance introduced by SVA and enhanced capacitive coupling in the energy concentration region in Fig 3, the circuit may exhibit resonance. This resonance can potentially create two additional finite transmission zeros(TZ), which can be utilized for signal suppression or notch filtering effects. Next, based on the novel coupling signal path design, a bandpass filter comprising five passband modes *f *1 ,*f *2 ,*f *3 ,*f *4 and *f *5 is constructed.

**Fig 4:**
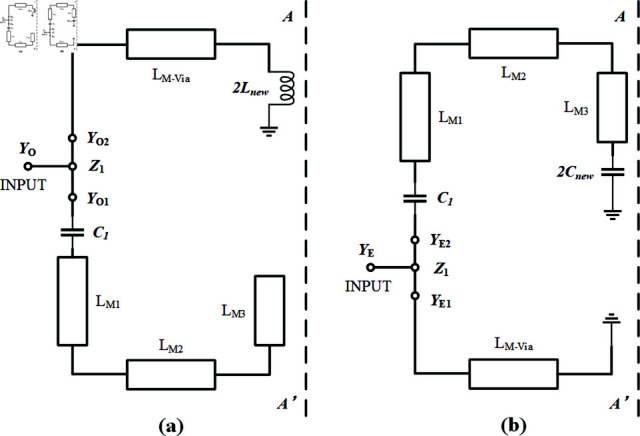
Simplified equivalent circuit diagram of SVA-ML-CPW. (a) Odd mode. (b) Even mode.

### 1.1 First passband mode *f *1 analysis

The first passband mode f1 is controlled by the signal path consisting of the SVA-ML-CPW as depicted in Fig 3, *C*1 represents the vertical coupling between microstrip lines and the gro. Due to the circuit’s inherent symmetry, an odd-even mode analysis method [20] is employed to analyze the circuit structure symmetrically about the centerline AA’ relative to the reference plane. The simplified circuits decomposed using the odd-even mode analysis are illustrated in Fig 4(a) for electric wall analysis and Fig 4(b) for magnetic wall analysis.

The simplified equivalent circuit in Fig 4 retains only half of the centrally symmetric paths from Fig 3, resulting in the new inductance *Lnew* and capacitance *Cnew* being twice as strong as those in Fig 3. When the microstrip line is configured in an open-circuit parallel arrangement, it exhibits parallel odd-mode admittance YO (YO1//YO2) and parallel even-mode admittance YE (YE1//YE2). Under resonance conditions, this is described by and 2:


$$\begin{array}{c} Y_{o}=Y_{o1}+Y_{o2}=0 \end{array}$$ (1)

$$\begin{array}{c} Y_{E}=Y_{E1}+Y_{E2}=0 \end{array}$$ (2)

Using transmission line theory [21], the admittance at each virtual node in Fig 4 is calculated. The admittance at node YO1 is derived using the formula for lossless terminated transmission lines with an open circuit load, as shown in . The admittance at node YE1 is obtained using the formula for lossless terminated transmission lines with a short circuit load, as presented in . The admittances at nodes YO2 and YE2 are further calculated using the formula for lossless terminated transmission lines with a load, as demonstrated in and 6.


YO1=−jY1 cot ⁡ βoLM−Via (3)

YO2=jY1(2ωOLnew+Y1 tan ⁡ βOLM)(Y1−2ωOLnewtanβOLM) (4)

YE1=jY1 tan ⁡ βELM−Via (5)

YE2=jY12ωeCnewY1 tan ⁡ βELM−12ωeCnewY1+ tan ⁡ βELM−Via (6)

Substituting the nodal –6 according to the circuit in Fig 4, the expanded formula for parallel odd-mode admittance and parallel even-mode admittance :


YO=jY1 [−cot ⁡ βOLM−via+2ωOLnew+Y1 tan ⁡ βOLMY1−2ωOLnew tan ⁡ βOLM] (7)

YE=jY1 [tan ⁡ βELM−via+2ωECnewY1 tan ⁡ βELM−12ωECnewY1+ tan ⁡ βELM−via] (8)

In and 8, *β*O and *β*E represent the propagation constants for odd-mode and even-mode respectively, which are ideal mathematical analyses assuming extreme conditions of open and short circuits in the circuit. *ω*O and *ω*E are angular frequencies at the resonant frequencies of the odd and even modes. Y1 denotes the characteristic admittance in the resonant region, and the characteristic impedance is Z1 = 1/Y1. Simultaneously satisfying –8, a second-order Taylor expansion is performed at a quarter wavelength *λ*/4 along the total length of the microstrip line (LM+LM-via) to eliminate trigonometric functions, yielding the relationships and 10:


$$\begin{array}{c} \frac{\pi }{2}-\beta_{O}\left(L_{M-via}+L_{M}\right)=2Y_{1}\omega_{O}L_{\text{new}} \end{array}$$ (9)

$$\begin{array}{c} \frac{\pi }{2}-\beta_{E}\left(L_{M-via}+L_{M}\right)=2Z_{1}\omega_{E}C_{\text{new}} \end{array}$$ (10)

Transform the propagation constants *β*O and *β*E for odd-mode and even-mode from and 10 using into the square root of the dielectric constant $\sqrt{\varepsilon_{r}}$ of the substrate:


$$\begin{array}{c} \beta =\frac{\sqrt{\varepsilon_{r}}}{c} \end{array}$$ (11)

Where *c* represents the speed of light in vacuum. From –11, the odd-mode angular frequency center 12 and even-mode angular frequency center of the new coupling path can be derived. illustrates the center frequency *f *1 of the new coupling path implementation, defined as the arithmetic mean of the odd-mode and even-mode angular frequencies.


$$\begin{array}{c} \omega_{O}=\frac{\pi }{4}\times \frac{1}{Z_{1}L_{\text{new}}+\left(L_{M-via}+L_{M}\right)\sqrt{\varepsilon_{r}}∕c} \end{array}$$ (12)

$$\begin{array}{c} \omega_{E}=\frac{\pi }{4}\times \frac{1}{Y_{1}C_{\text{new}}+\left(L_{M\text{-via}}+L_{M}\right)\sqrt{\varepsilon_{r}}∕c} \end{array}$$ (13)

$$\begin{array}{c} f_{1}=\frac{\omega_{O}+\omega_{E}}{4\pi } \end{array}$$ (14)

### 1.2 Analysis of additional passbands

**Fig 5:**
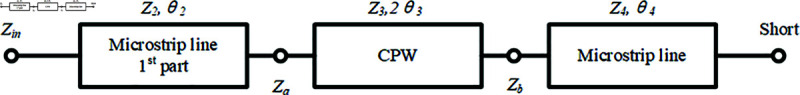
Simplified Equivalent Circuit Diagram of ML-CPW Signal Path.

The second, third, fourth, and fifth passband modes (*f *2 ,*f *3 ,*f *4 and *f *5) achieve higher frequency passbands with lower required electrical lengths compared to f1, governed by the ML-CPW signal path depicted in Fig 3. Extract the simplified equivalent circuit diagram of the ML-CPW from Fig 3 as depicted in Fig 5.

Fig 5 simplifies the half-wavelength resonant cavity of ML-CPW into an analysis of three-segment characteristic impedances (Z2-Z3-Z4). The microstrip line’s first and third segments are symmetric, exhibiting network reciprocity such that Z2 = Z4 and *β*2 = *β*4. Furthermore, interchange analysis between source and load ends is feasible at both ports. Currently, the left end is designated as the input impedance terminal, connecting the three-segment characteristic impedances ultimately to a short-circuited backplane, thereby making the right end the short-circuited load terminal. Specifically, CPW input impedance is denoted as Zin, with Z2 representing the characteristic impedance of the first and third segments, each having an electrical length of *β*2. The second segment exhibits a characteristic impedance of Z3 with an electrical length of *β*3, and the relationship among the electrical lengths of the three segments satisfies *β*2 ≈ ≈ 4. Each segment of the transmission line’s input impedance is analyzed separately, assuming the characteristic impedances of the two segments connecting at node in Fig 3 are Za and Zb, respectively. –17 provide the characteristic impedances of each node.


Zin =Z2Za+jZ2 tan ⁡ θ2Z2+jZa tan ⁡ θ2 (15)

Za=Z3Zb+jZ3 tan ⁡ θ3Z3+jZb tan ⁡ θ3 (16)

Za=jZ4 tan ⁡ θ4 (17)

Integrating –17, the total input impedance Zin of the three-section characteristic impedance is obtained.


Zin =Z22(tan ⁡ θ2+Z2Z3 tan ⁡ θ3)(Z2Z3− tan ⁡ θ2 tan ⁡ θ3)Z2Z3(1− tan ⁡ 2θ2)(1− tan ⁡ 2θ3)−2(1+(Z2Z3)2 tan ⁡ θ2 tan ⁡ θ3) (18)

To achieve sufficiently strong bandpass modes and form a passband, ideally, the input impedance Zin should be zero for maximum resonance. Therefore, the numerator in should be zero, as given in .


Z2Z3− tan ⁡ θ2 tan ⁡ θ3=0 (19)

Based on Eq (1.16), the total electrical length of the half-wavelength resonant cavity can generate multiple harmonic modes to construct a passband.


$$\begin{array}{c} \theta_{L}=\theta_{2}+2\theta_{3}+\theta_{4} \end{array}$$ (20)

Clearly, by adjusting the three-section characteristic impedances and electrical lengths ≈ 2 and ≈ 3 to satisfy and 20 simultaneously, four sufficiently spaced and strong resonance modes *f *2 ,*f *3 ,*f *4 and *f *5 can be obtained. Therefore, –24 are derived:


f2=2πθ2f3=2πarctan ⁡ Z2Z3f3 (21)

$$\begin{array}{c} f_{3}=\frac{c}{2\pi \theta_{L}\sqrt{\varepsilon_{r}}} \end{array}$$ (22)

f4=2π(π−θ2)f3=2π(π− arctan ⁡ Z2Z3)f3 (23)

$$\begin{array}{c} f_{5}=2f_{3}=\frac{c}{\pi \theta_{L}\sqrt{\varepsilon_{r}}} \end{array}$$ (24)

By normalizing with the passband mode *f *3 as the center frequency in the calculation of half-wavelength resonant cavities, it was found possible to achieve four resonant modes: *f *2 ,*f *3 ,*f *4 and *f *5. The passband mode distribution is controlled by the ratio Z2/Z3, and when Z2/Z3 equals 2, the distribution is optimized. The electrical characteristics of the first CPW structure should be Z2 = 53 ohms, with an electrical length of 36 degrees, corresponding to physical characteristics of CPW1 = 3 mm, and G1 = 4 mm. The impedance of the second CPW structure should be Z2 = 27ohms, with an electrical length of 74 degrees, corresponding to physical characteristics of CPW2 = 6 mm, W1 = 2.6 mm, and G2 = 1.5 mm. The slotline width G2 is further influenced by the notch structure, which will be discussed in Sect 2.1.

**Fig 6:**
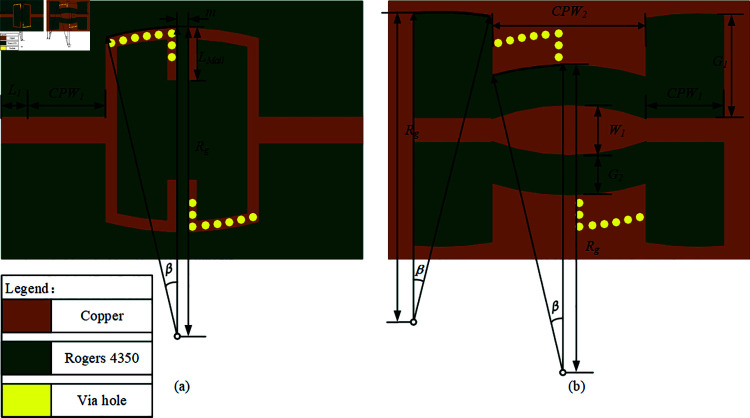
Simulation model of SVA-ML-CPW. (a)Top view. (b)Bottom view.

Higher-order resonance modes above *f *5 become parasitic passbands affecting the passband. These parasitic passbands will impact the high-frequency transmission zeros in the ML-CPW structure [15,19,22], which are crucial for achieving high selectivity in the stopband. Therefore, in the subsequent Sect 2.3, a defect structure will be introduced to suppress these parasitic passbands. Combining the distribution patterns of these five passbands, the simulated model of SVA-ML-CPW penta bandpass modes filter designed based on a novel coupling circuit is presented in Fig 6. Upon examining the simulation model, it can be observed that some copper-free dielectric substrate areas are reserved at the top and bottom of the device for integrating the five notch filter structures. The detailed procedure is presented in Sect 2, "Five Notch Filter Designs for Signal Suppression. Therefore, in Fig 6, the slotline width G1 is maximized without affecting the passband to accommodate the notch structure. A larger slotline width also reduces the coupling between the notch structure and the CPW ground. The feedline length L1 should be selected carefully; if L1 is too long, it increases costs, and if too short, it may affect soldering. Thus, an appropriate length of L1 = 1 mm is chosen. Additionally, curving the microstrip line and CPW helps reduce the generation of harmonics within the passband, with the curvature parameters set to Rg = 11.5 mm and β=14^∘^. Finally, the parameter m will be discussed in the context of weak coupling design, where the optimal solution will be provided.

The simulated filter adopts a three-layer vertical board structure with Rogers 4350 as the substrate material having a dielectric constant of 3.66. The substrate thickness is standardized at 0.508 mm, and the overall dimensions are 10.8 × 14mm^2^. Both modeling and electromagnetic simulations in this study utilize FEM software ANSYS Electronics 2022.2 HFSS. To summarize, the design parameters for Fig 6 are: L1 = 1 mm, Rg = 1 mm, CPW1 = 3 mm, CPW2 = 6 mm, G1 = 4 mm, G2 = 1.5 mm, *β* = 14°.

### 1.3 Weak coupling analysis

Integrating the passband mode analysis from Sects 1 and 2 with the establishment of the simulation model in Fig 6, we examine four primary passband mode frequency control parameters for the filter under weak coupling: the number of via array elements, total length of coupled microstrip lines LMall, microstrip coupling distance m, and the width of the second CPW section W1.

**Fig 7:**
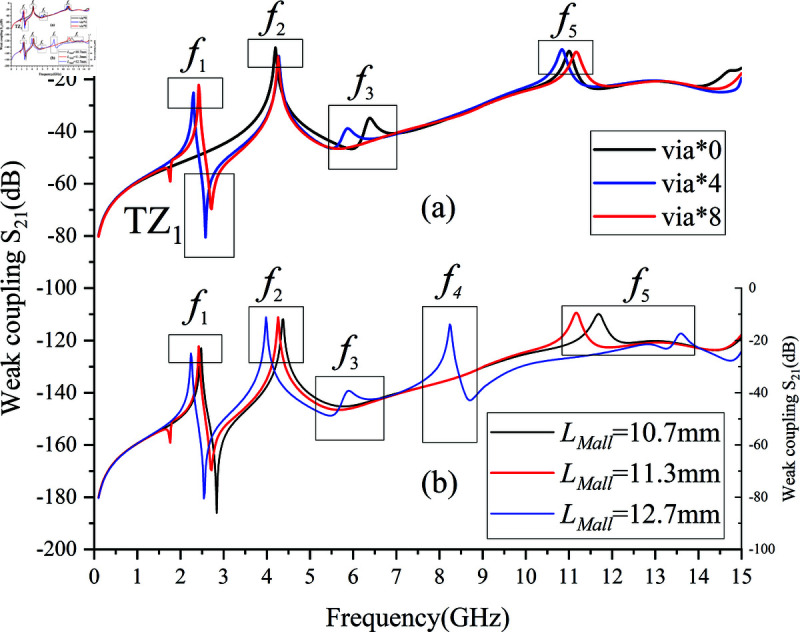
Effect of control parameters on passband mode. (a)Number of via hole elements. (b)Total length of coupled microstrip line *L_M_all.*


Fig 7(a) demonstrates the impact of varying the number of via hole array elements on the frequencies of different modes under weak coupling. The number of via hole array elements directly affects the inductance introduced as shown in Fig 3. When the number of via hole array elements is zero, the SVA-ML-CPW path is lost, leading to the disappearance of the first mode f1.As the number of via array elements increases, the SVA-ML-CPW path is generated, and both mode f1 and and transmission zero TZ1 appear.Overall, Overall, the frequencies of all five modes slightly shift to higher frequencies as the number of via array elements increases. With the number of via hole array elements fixed at 8, we next analyze the influence of the total length of coupled microstrip lines LMall on the frequencies of each mode as depicted in Fig 7(b), where provides the composition of LMall.

$$\begin{array}{c} L_{M}all=L_{M}1+L_{M}2+L_{M}3+L_{M}-via \end{array}$$ (25)

Observing Fig 7(b), the total length of coupled microstrip lines LMall significantly affects the overall electrical length, making it the most influential among the four control parameters. As LMall increases, modes *f *1 and *f *2 shift towards lower frequencies, while *f *3 and *f *4 exhibit prominent peaks. However, *f *5 weakens, and its center frequency moves from 11.1 GHz to around 15.5 GHz. The low intensity and separation from *f *4 make *f *5 susceptible to high passband insertion loss beyond 10 GHz. To maintain low insertion loss in the high-frequency passband, *f *5 needs to be strengthened. Therefore, in most weak coupling modes, the peaks of *f *3 and *f *4 are not prominently displayed.

**Fig 8:**
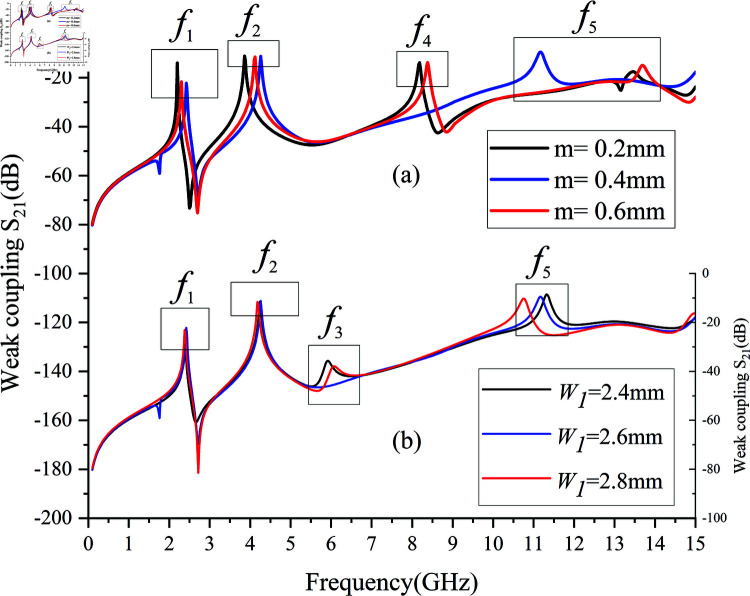
Effect of control parameters on passband mode. (a) Microstrip coupling distance m. (b) Width of the second CPW section W1.

Analysis of two additional control parameters’ effects on passband mode distribution follows. Fig 8(a) demonstrates how altering microstrip line coupling distance *m* influences mode frequencies under weak coupling, directly impacting the capacitance introduced in Fig 3. Frequencies *f *1, *f *2, and *f *4 vary with *m*, while *f *5 is significantly affected, moving further from *f *4 and weakening regardless of *m*’s size, hence the optimal distance being 0.4mm. Fig 8(b) explores changing the width W1 of the second CPW section, adjusting characteristic impedance Z3 depicted in Fig 5. By adjusting the parameter W1, the impedance matching of the stepped impedance structure is modified [23], thereby achieving the desired operating center frequency. Consistent with passband design principles in Sect 2, the first mode *f *1 remains largely unaffected, whereas modes *f *2, *f *3, *f *4, and *f *5 gradually shift towards lower frequencies with increasing W1, aligning with .

Based on the previous discussion, the five ideal passband mode frequency centers designed by the in Sect 1 and –24 in Sect 2 are *f *1 = 2.35 GHz, *f *2 = 3.57 GHz, *f *3 = 5.95 GHz, *f *4 = 8.35 GHz, and *f *5 = 11.9 GHz. The number of via hole array elements is determined to be 8, the total length of the coupled microstrip line LMall = 11.3 mm, the coupling distance of the microstrip line m = 0.4 mm, and the second segment CPW width W1 = 2.6 mm. The actual passband mode frequency centers obtained from the weak coupling mode are *f *1 = 2.42 GHz, *f *2 = 4.20 GHz, *f *3 = 5.93 GHz, *f *5 = 8.26 GHz, and *f *6 = 11.8 GHz.

From the perspective of the transfer function, the five ideal center frequencies of the passbands correspond to five transmission poles. The SVA-ML-CPW filter structure utilizes these five transmission poles to achieve the bandpass filter, with their allocation for passband adjustment detailed in Sect 1.3 on weak coupling design. The filter inherently includes two transmission zeros: one for implementing a notch around 4 GHz and another for enhancing the stopband near 15 GHz. Additionally, the notch structures introduced in Sect 2 add four transmission zeros, enabling multi-notch functionality on top of the bandpass filter. In summary, to achieve the five-notch wideband filter as outlined in the manuscript, the design requires not only the five transmission poles and two inherent transmission zeros but also the addition of four extra transmission zeros. The significant deviation of *f *2 is due to the transmission zero point TZ1 generated by the circuit in Fig 3. In the next section, Sect 2, methods such as utilizing the transmission zero point TZ1, proposing a double hairpin structure, and improved structures will be used to achieve signal suppression for interference signals in the five 5G/6G commercial communication frequency bands.

## 2 Five notch filter designs for signal suppression

Due to the complexity of the modern wireless transmission environment, it is necessary to suppress the interference caused by signals from popular commercial frequency bands to protect communication in the S/C/X/KU band. Meanwhile, to adapt to the miniaturization trend of modern wireless communication systems, this manuscript provides various notch structures integrated into microstrip bandpass filters for suppressing 5G/6G frequency bands. Additionally, in Sect 2.2, this manuscript proposes a compact embedded dual-bandstop hairpin structure with a tunable center frequency for the C band.

To suppress interference from 5G base station services [24], 5G WIFI bands [25], the newly implemented 6G IMT system, point-to-point fixed wireless systems, and the Starlink mega-constellation satellite network [26] in the S/C/X/KU passband, the following methods will be used for signal suppression:

1. Utilizing the transmission zero point created by the SVA-ML-CPW signal path to suppress 5G base station service signals (3.7–4.0 GHz) in the N77 frequency band [27].

2. Using the proposed dual-bandstop hairpin stub notch structure to suppress two frequency bands: the U-NII-2 protocol of 5G WIFI (5.250–5.725 GHz) and the 6G IMT system signals (6.425–7.125 GHz).

3. Employing open-loop resonators to suppress the broadband signals of the point-to-point fixed wireless system for satellite TV communication (10.7–11.7 GHz).

4. Applying the inverted S-shaped EBG structure to suppress Starlink user uplink signals (12.75–13.25 GHz).

### 2.1 First notch filter design

By adjusting and optimizing the filter’s dimensional parameters, the filter achieves a passband in the S/C/X/KU band while obtaining two resonant transmission zero points. The upper transmission zero point TZ1 is used to create the first notch *f *A to suppress 5G base station service signals in the 3.7–4.0 GHz range, while the lower transmission zero point TZ2 is used to achieve high selectivity of the filter.

Clearly, the control of transmission zero TZ1 can be achieved by adjusting the parameters L_MALL_ and G_2_ of the SVA-ML-CPW structure. However, two design prerequisites must be considered before achieving controllability of TZ_1_: 1) In structures like CPW GROUND, the width W_1_ of the signal conductor in CPW GROUND significantly affects the impedance, whereas the ground gap between the conductor and ground planes, represented by the slotline width G_2_ in this paper, has a relatively smaller impact on impedance. 2) Given the constraints of the impedance ratio Z_2_/Z_1_ in the stepped impedance described in Sect 1.2, W1 should be minimally altered. Therefore, by modifying G_2_, the capacitive coupling Cnew in the SVA-ML-CPW path of Circuit Fig 4 can be influenced, thereby allowing control over the center frequency,notch frequency range, and selectivity of TZ_1_.

**Fig 9:**
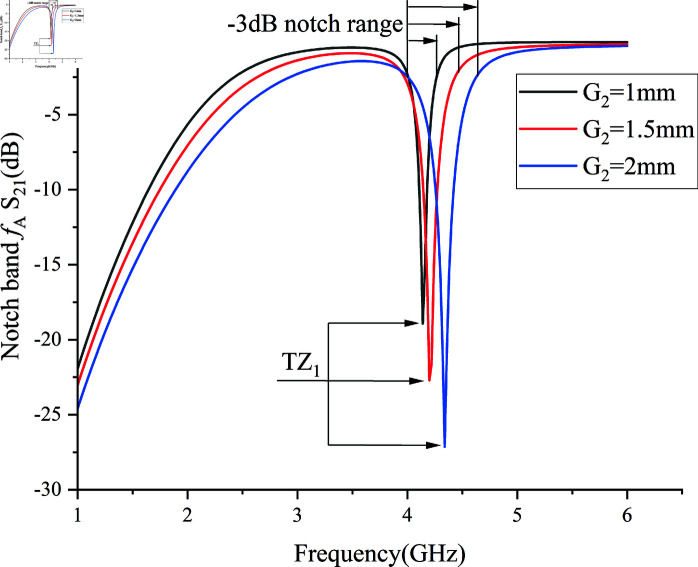
Impact of slotline width variation G2 on the first notch band.

From a theoretical perspective, as G_2_ increases, the capacitive coupling weakens, leading to a reduction in the strength of the passband mode f1 while the strength of the transmission zero TZ_1_ generated by circuit resonance remains unchanged. By observing the effect of varying G_2_ on the notch bands in Fig 9, three key notch band parameters—center frequency,notch frequency range, and selectivity—are affected as G2 increases: 1) The notch center frequency shifts slightly towards higher frequencies, attributed to the reduced electrical length of the SVA-ML-CPW path as the slotline width increases. 2) The notch bandwidth expands from 0.22 GHz to 0.53 GHz, the –3 dB passband above 5 GHz remains unaffected, while the 2–4 GHz –3 dB passband narrows. 3) Notch selectivity decreases from 0.32 to 0.3, with the –10 dB suppression rate dropping from –87 dB/GHz to –35 dB/GHz, due to the weakened passband mode, which reduces the shape factor of the transition from the –3 dB passband to the –10 dB stopband.

**Fig 10:**
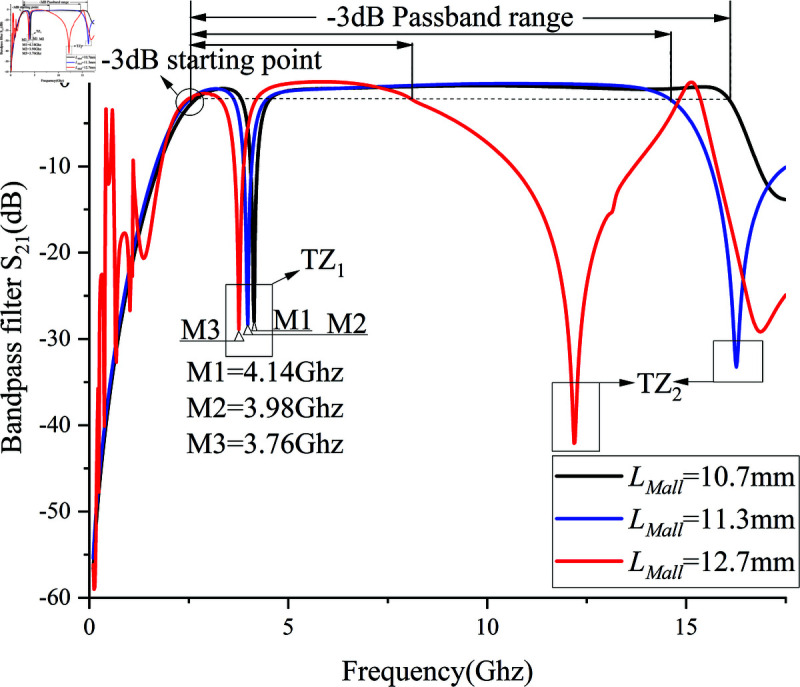
Filter passband and transmission zero points.

Subsequently, by adjusting the most influential control parameter, the total length of the coupled microstrip line LMall, the filter’s passband and transmission zero point positions are optimized, as shown in Fig 10.The insertion loss of the prototype bandpass filter in the figure is only –0.41 dB. First, considering the appropriate –3 dB passband range, the initial frequency points of the three passbands, marked in Fig 10 as –3 dB starting points, show minimal positional change with varying length LMall. However, the resulting –3 dB passband ranges differ significantly. As the length LMall increases from 10.7 mm to 12.7 mm, the –3 dB passband range rapidly decreases from 13.7 GHz to 6.14 GHz. When LMall is too small, harmonic distortions appear in the low-frequency passband, and transmission zero point TZ2 shifts to lower frequencies, resulting in a rapid reduction of passband width. Conversely, when LMall is too large, TZ2 disappears, leading to a loss of high selectivity at the filter’s edge. Integrating observations from Figs 9 and 10, it is evident that altering the total length LMall of the microstrip line can adjust the center frequency of TZ1 without affecting the notch band’s frequency range or selectivity. However, excessively large or small lengths may negatively impact the overall –3 dB passband range. In contrast, changing the slotline width G2 simultaneously adjusts the center frequency of TZ1 as well as the notch band’s frequency range and selectivity.

Therefore, G2 = 1.5 mm, LMall = 11.3 mm was chosen, with corresponding capacitance and inductance values of Lnew = 1.4 nH and Cnew = 0.08 pF. This configuration allows the filter to achieve an exceptionally wide passband and low insertion loss, while maintaining two suitable transmission zero points: TZ1 at 3.98 GHz and TZ2 at 16.82 GHz. Additionally, TZ1 has a transmission zero point strength of –29.38 dB. Consequently, TZ1 at 3.98 GHz is used to design the first notch filter *f *A for the 3.7–4.0 GHz band.

After completing the design of *f *A, it is necessary to re-evaluate the impact of *f *A on the passband. Observing Fig 10, the bandpass filter with LMall = 11.3 mm achieves a low insertion loss (–1 dB) passband range of 2.44–15.32 GHz. However, within the 4.16–6.76 GHz passband, the insertion loss exceeds –1 dB. This is to ensure the resonance and high strength of transmission zero point TZ1 but results in a weakened signal path strength for the passband. Therefore, a dual-band notch paperclip structure is designed next in this paper to enhance CPW coupling and reduce the passband loss caused by *f *A.

### 2.2 Second and third notch filter designs

As the current development trend of RF passive devices is to achieve excellent selectivity while maintaining miniaturized dimensions, this manuscript proposes new notch structures that utilize the internal space of the filter, without requiring external connections or additional structures on the dielectric substrate. Therefore, the dual narrowband notch paperclip open-circuit structures, symmetrically embedded in the CPW slot lines proposed in this manuscript, can suppress two 5G/6G commercial bands in the C-band: 5.250–5.725 GHz and 6.425–7.125 GHz. The design targets for the two notch center frequencies are *f *B = 5.48 GHz and *f *C = 6.78 GHz, respectively.

**Fig 11:**
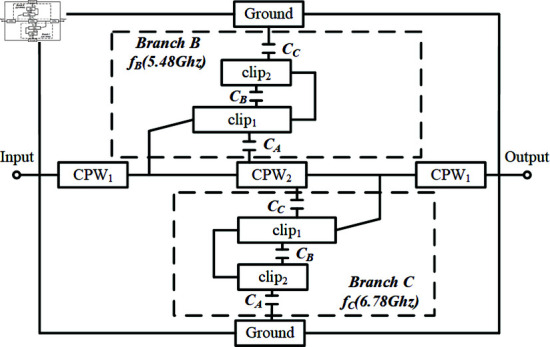
Topology of the dual narrowband notch paperclip structure.

Branch B and Branch C are placed adjacent to the region with the strongest inductive coupling in the new coupling path and within the slot line G2 where capacitive coupling in the microstrip-CPW is strongest, providing two different notch paths for the signal, thereby offering the potential to form multiple notches. To ensure sufficient strength of the low-frequency notch *f *B, the longer branch (Branch B) is placed near the input end, while the shorter branch (Branch C) is positioned near the output end, as shown in Fig 11. Here, CA represents the coupling of the paperclip branch clip1 to the second section of CPW, CB denotes the self-couple of the paperclip structure, and CC indicates the coupling of the paperclip branch clip2 to the CPW ground. The capacitive couplings CB and CC can address the high insertion loss in the passband caused by the transmission zero point discussed in Sect 2.1, while the self-couple CB enhances the notch strength. The notch center is determined by the total electrical length of clip1 and clip2. The topology of the dual narrowband notch paperclip structure is shown in Fig 11.

**Fig 12:**
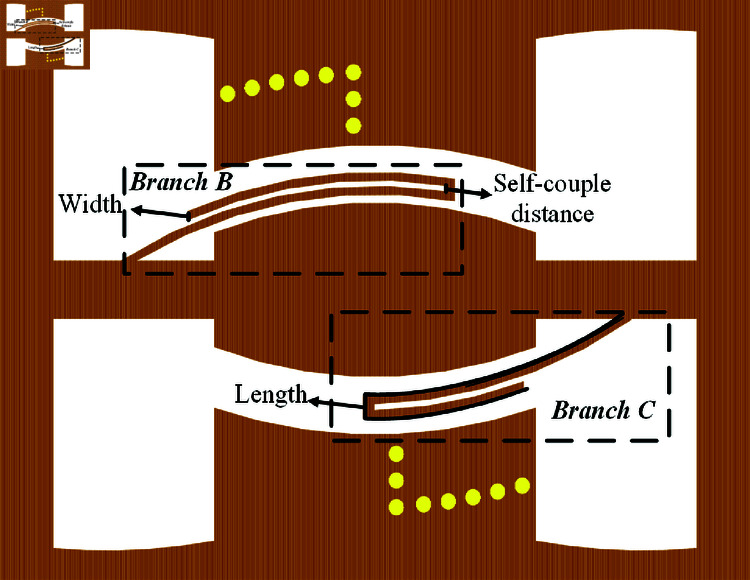
Dual narrowband notch paperclip structure: Branch B and Branch C.

Compared to the direct connection of branches to the feed end as described in [28], in Fig 12, the branches are connected to the first section of CPW1. This approach effectively reduces the impact on the passband flatness around the notch, but correspondingly, the notch strengths of *f *B and *f *C are somewhat reduced. Therefore, the self-couple CB generated by the microstrip lines at both ends of the paperclip shape is used to enhance the notch strength. Next, based on Fig 11, the simulation model of the dual narrowband notch paperclip structure is presented in Fig 12.

**Fig 13:**
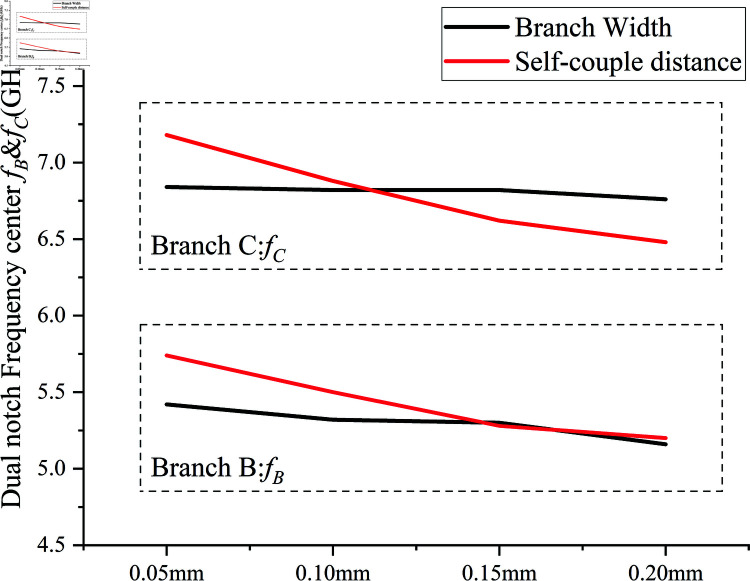
Effect of branch width and self-couple distance on notch center frequencies *f *B and *f *C.

It is evident that adjusting three parameters of the paperclip model—the total length of the branches, the width of the branches, and the self-couple distance can change three indicators of the double notch band: center frequencies fB and fc, notch frequency range, and selectivity. First, we observe the effect of changing the branch thickness and self-couple distance on the dual notch center frequencies, as shown in Fig 13. When the branch width and self-couple distance increase, the center frequencies *f *B and *f *C. tend to shift towards lower frequencies. However, the increase in branch width causes only a slight shift in *f *B and *f *C, whereas the increase in self-couple distance results in a more significant shift.

**Fig 14:**
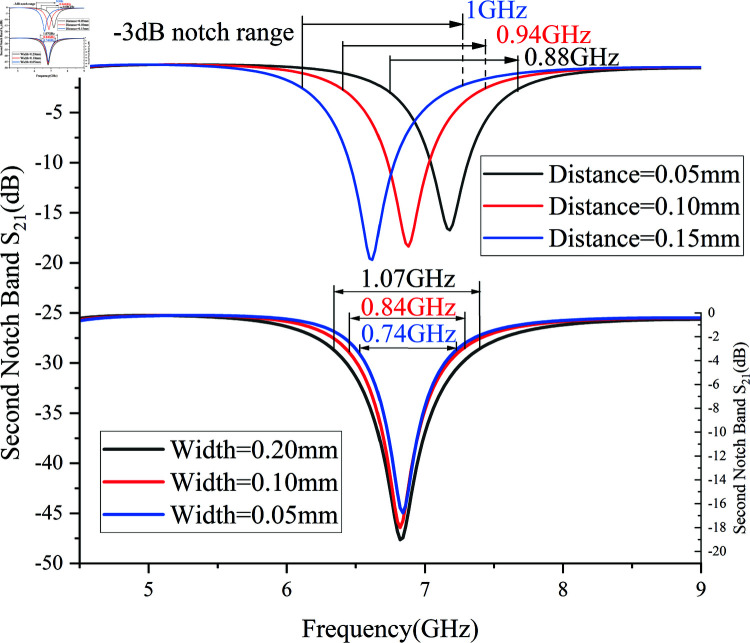
Impact of branch width and self-couple distance on the notch band.

The following analysis examines the impact of varying branch width and self-couple distance on the frequency range and selectivity of the notch band, as shown in Fig 14. When the self-couple distance is increased from 0.05 mm to 0.15 mm, the –3 dB notch frequency range decreases from 1 GHz to 0.88 GHz, while the –10 dB selectivity rapidly increases from 0.22 to 0.32. Conversely, when the branch width is increased from 0.05 mm to 0.20 mm, the –3 dB notch frequency range expands from 0.74 GHz to 1.07 GHz, but the –10 dB selectivity slightly decreases from 0.32 to 0.30. Therefore, when determining the self-couple distance, it is important to consider the trade-off between notch frequency range and selectivity. Adjusting the notch band’s frequency range without significantly altering selectivity can be achieved by varying the branch width(Adjusting the frequency range of the notch band with minimal impact on selectivity can be achieved by varying the branch width.).

**Fig 15:**
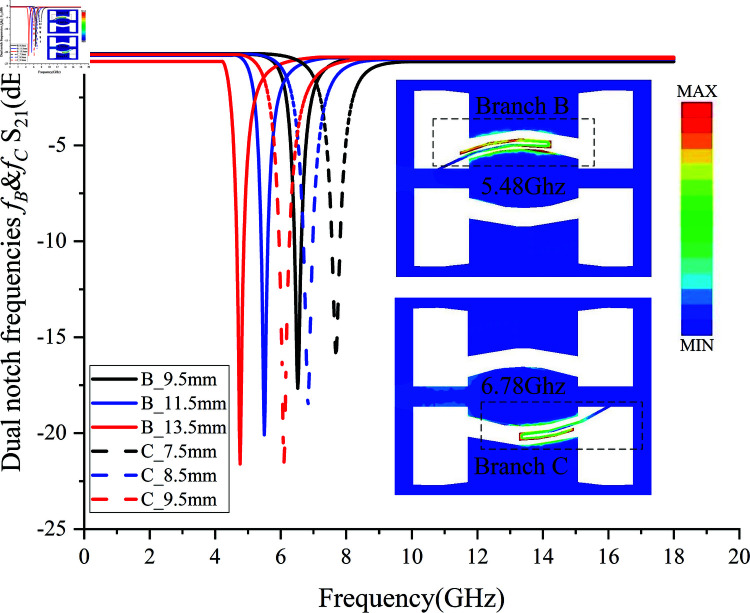
Electric field distribution and the effect of total branch length on notch center frequencies *f *B and *f *C.

Since clip1 and clip2 have considerable adjustable electrical lengths, the total length of the branches has a greater adjustable range compared to the branch width and self-couple distance when controlling the notch center frequencies. This allows for better control of the center frequencies *f *B and *f *C. Now, by fixing the branch width at 0.15 mm and the self-couple distance at 0.10 mm, and adjusting the total length of the branches, *f *B is set to 5.50 GHz and *f *C to 6.82 GHz. Observing Fig 15, we analyze the effect of changing the total length of the dual paperclip branches on the dual notch center frequencies *f *B and *f *C.

Fig 15 shows that as the length of the dual paperclip branches increases, the dual notch center frequencies shift significantly towards lower frequencies. Within the 4–8 GHz range, the peak values in the C-band exceed –15 dB, and the –10 dB suppression rate of the dual branches surpasses –38.89 dB/GHz, indicating excellent suppression performance. As the total length of the branches increases, the self-couple strength of the branches also increases, as evidenced by the enhanced CB strength in Fig 10, which results in an increase in notch depth to exceed –20 dB.

The right side of Fig 15 also shows the current density distribution in the paperclip branches at the operating frequencies of 5.48 GHz and 6.78 GHz. At these frequencies, the current is concentrated in the paperclip branches and does not flow to the output, thereby achieving the notch function.

Compared to the physical lengths of 27.33 mm and 22.14 mm required for the half-open short-circuited lines at 5.488 GHz and 6.775 GHz frequencies, respectively, the use of dual paperclip branches can effectively reduce the total length by 133% and 161%. Compared to the physical lengths of 26.06 mm and 21.10 mm required for directly coupled meandering lines at 5.488 GHz and 6.775 GHz frequencies, respectively, the dual paperclip branches can effectively reduce the total length by 136% and 149%. When the branch width is set to 0.15 mm and the self-coupling distance to 0.10 mm, with dual branch lengths of 11.5 mm and 8.5 mm, the second and third notch bands are precisely positioned at 5.25-–5.75 GHz and 6.425—7.125 GHz, respectively. With its precise notch band control, this filter demonstrates a significant advantage in accurately defining the notch band frequency range, achieving a maximum frequency range accuracy of 0.001 GHz. This high-precision control not only enhances the filter’s interference suppression capability in complex signal environments but also provides a quantitative benchmark for the accuracy parameters in the comparison table.

The use of paperclip branches for notch filter structures not only eliminates the need for external expansion of the resonator but also allows for placement within the CPW slot line, achieving a smaller overall volume that facilitates miniaturization. Additionally, it provides sufficient notch depth, rapid notch suppression speed, and excellent design convenience. The dual-band notch paperclip structure is highly suitable for designing flexible notch centers to suppress any narrow-band interference signals in the C-band.

So far, this paper has implemented a three notch filter. Before advancing to the five notch filter, two issues related to higher-order harmonics induced by the notch structures need to be addressed: Will the paperclip structure excite higher-order harmonics? Will the excited higher-order harmonics affect the passband? These higher-order harmonic issues will be discussed in the latter part of Sect 2.2.

Firstly, the first notch structure *f *A is based on resonances in the SVA-ML-CPW signal path. Due to the influence of multiple high-intensity passband modes in the weak coupling design discussed in Sect 1.3, higher-order harmonics are not activated. On the other hand, the second and third notch structures *f *B and *f *c extend beyond the signal path. If a standalone notch structure does not have sufficient passband mode influence, higher-order harmonics may be excited. provides the approximate higher-order harmonic frequencies for an open-circuited transmission line.


f=π2×ntan ⁡ βl (26)

**Fig 16:**
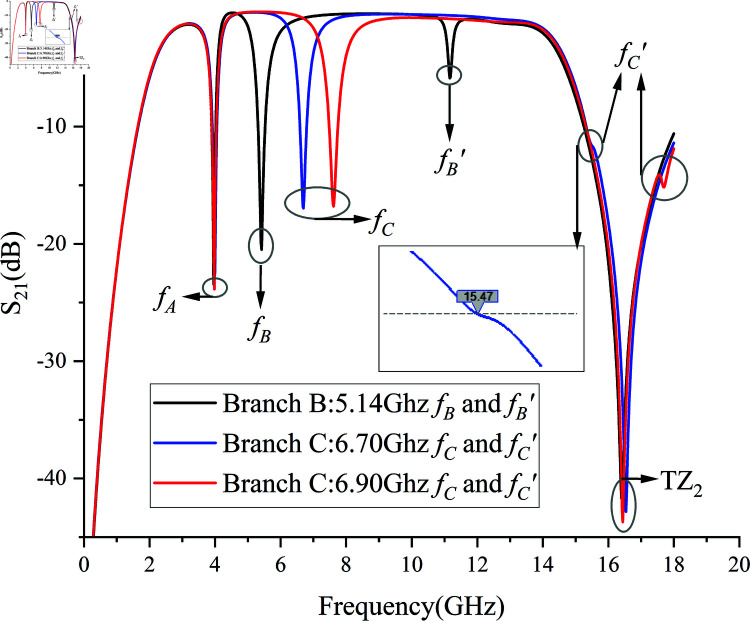
Second-Order Harmonics *f *B*′* and *f *C*′* of *f *B and *f *C.

Here, *l* represents the transmission line length, *β* is the transmission line propagation constant, and *n* is the harmonic order (*n* = 1,2,3,…). According to , the next higher-order harmonic (with *n* = 2) for the notch structures *f *B and *f *c is expected to appear at integer multiples of half-wavelength frequencies. Specifically, the second-order harmonics for *f *B and *f *c are predicted to occur around frequencies of 10.96 GHz and 13.56 GHz, respectively. The double paperclip notch structure was integrated into the filter simulation model, and the higher-order harmonic states were observed in Fig 16.

Observation of Fig 16 reveals the second-order harmonic frequency centers of *f *B and *f *C. When the notch frequencies *f *B and *f *C are 5.14 GHz, 6.70 GHz, and 6.90 GHz respectively, the attenuation for all three exceeds –20 dB. The second-order harmonic of *f *B at 5.14 GHz appears at 11.14 GHz. The second-order harmonics of *f *C appear at 15.47 GHz and 17.7 GHz, respectively. *f *C*′* is close to the passband edge, but due to the protection of the high-selectivity transmission zero TZ2 at the passband edge, *f *C*′* has little effect on the passband. During the optimization of the five notch filter integration, the second-order harmonic *f *C*′* can be shifted to frequencies outside the passband to protect the passband or combined with TZ2 to enhance selectivity. However, the impact of the second-order harmonic *f *B*′* on the passband cannot be ignored, thus its strength needs to be minimized. The strength of *f *B*′* originates from the capacitive couplings CA and CC in Fig 16. Therefore, theoretically, *f *B*′* can be eliminated by adjusting the position of the microstrip line and the self-couple distance.

**Fig 17:**
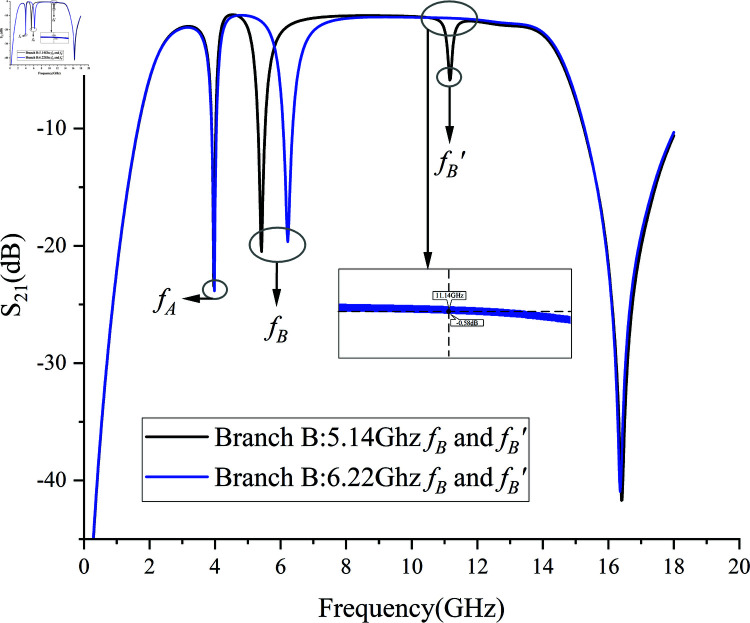
The appearance and disappearance of *f *B^′^.

When adjusting branch B to center in slotline G2, minimizing the strengths of CA and CC, and setting the self-couple distance to 0.02 mm, *f *B shifts from 5.14 GHz to 6.22 GHz without changing the length of branch B. Although branch B loses a considerable amount of electrical length, the disappearance of *f *B*′* is achieved, while maintaining an insertion loss of less than –1 dB across the 6–15 GHz passband. The appearance and disappearance of *f *B*′* are shown in Fig 17.

By comparing the 4–8 GHz passbands in Fig 10 and Fig 16, it is evident that the insertion of the double-pinned structure reduces the high insertion loss caused by TZ1, decreasing the 4–8 GHz passband insertion loss from –1.4 dB to –0.2 dB. However, due to the presence of the higher-order harmonic *f *B*′*, readers need to select the appropriate filter design based on their specific needs:

1) If low insertion loss in the passband is the primary goal, the 4–6 GHz notch *f *B*′* can be omitted;
2) If the notch *f *B is required but without the generation of the higher-order harmonic *f *B*′*, a larger waveguide size for the entire board is necessary to compensate for the electrical length loss of the notch *f *B, and a self-couple distance of 0.02 mm requires very high PCB manufacturing precision;
3) If there is still a need for a notch in the 8–15 GHz range, *f *B*′* can be retained, converting the originally harmful *f *B*′* into a beneficial enhancement for the 8–15 GHz notch structure. In the following Sect 2.3, an 8–15 GHz notch structure will be added based on the double-pinned structure discussed in Sect 2.2.

### 2.3 Fourth and fifth notch filter designs

After addressing the issue of interference from terrestrial commercial communication signals in S/C/X/KU band wireless systems, the focus of the filter notch functionality design shifts to addressing interference from satellite commercial communication signals. The evolution from traditional satellite television communication to the rapidly developing low Earth orbit mega-satellite network communication has heightened the requirements for notch filter functionality in ground-based reception systems. To meet these demands, this study further upgrades the three-notch filter to a five notch filter, enabling it to filter out common satellite frequency bands: the fixed wireless system (FWS, 10.7–11.7 GHz) used for satellite television communication and the uplink signals of the Starlink system (12.50–13.25 GHz).

**Fig 18:**
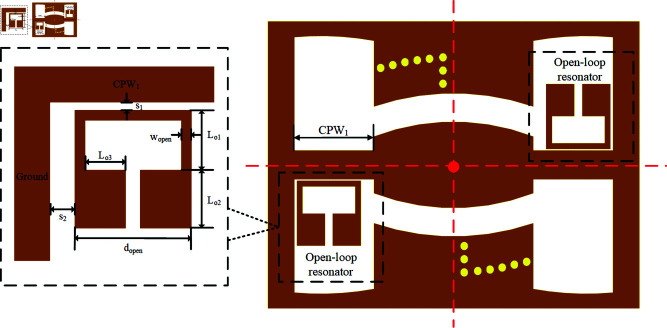
The open-loop resonator.

Based on the overall structural symmetry, the fourth notch *f *D is realized by placing an open-loop resonator [29] in slotline G1 for direct coupling with CPW1, with two symmetrically placed open-loop resonators to ensure notch strength. The fifth notch *f *E is constructed by incorporating an inverted S-shaped EBG structure at the output end of the coupled microstrip line. Finally, a DGS structure is added to enhance stopband performance. The structure and parameters of the open-loop resonator are shown in Fig 18.

This study utilizes the frequency dispersion generated by the open-loop resonator to achieve a wide band rejection with a notch width exceeding 1 GHz. Compared to the wide stopband generated at 3 GHz in [29], this paper places the open-loop resonator on the broadside coupling between the microstrip line and CPW1. The resonator is symmetrically placed at the slotline G1 on each side of CPW1 and CPW3, using two identical structures to achieve stronger notch intensity. provides the prediction of the total length of the open-loop resonator:


$$\begin{array}{c} \lambda_{l}oop=\frac{v_{p}}{f_{l}oop\sqrt{\varepsilon_{r}}} \end{array}$$ (27)

**Fig 19:**
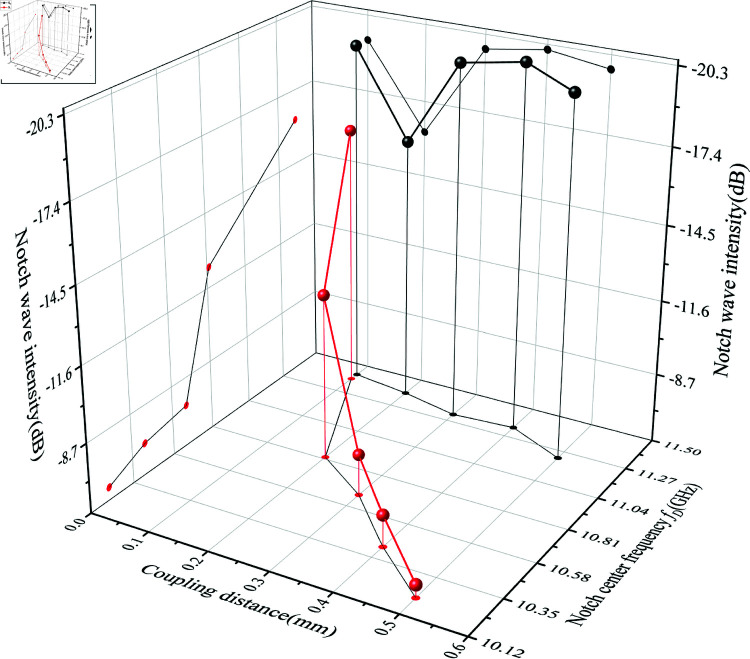
Impact of coupling distances S1 and S2 on the notch center frequency and suppression depth of the open-loop resonator.

In , vp represents the speed of light in a vacuum, *f * is the target center frequency of the open-loop resonator at 11.2 GHz, and *λ*loop is the target wavelength of the open-loop resonator. To minimize the impact of the open-loop resonator on the passband and other notch structures, a small-sized open-loop resonator with a total length of 9.5 mm is fabricated. The notch center frequency *f *D is then adjusted to 11.2 GHz using the direct coupling method. Reference [30] clearly states that the actual response of the resonator is not only related to the reactance values caused by the device’s size and material but also to its frequency. Evidently, with the total length restricted, the coupling distances S1 and S2 are crucial for the capacitive coupling between the open-loop resonator and CPW1, which adjusts the resonance center frequency fD. Next, by varying the coupling distances S1 and S2 in Fig 18, we observe changes in the center frequency and suppression depth of the open-loop resonator, as shown in Fig 19.

In Fig 19, the coupling distances S1 and S2 are the independent variables, while the notch depth and notch center frequency *f *D are the dependent variables. Fig 17 shows that changes in the coupling distance S1 have a greater impact on the performance of the open-loop resonator compared to changes in S2. This is because S1 is closer to the signal transmission path than S2. When both S1 and S2 are less than 0.5 mm, the center frequency of the open-loop resonator shifts above 10.12 GHz. As the coupling distance S1 decreases, the resonance center frequency *f *D and the notch depth increase significantly; however, as the coupling distance S2 decreases, the resonance center frequency *f *D and the notch depth increase only slightly.

Compared to the previous three notches, this notch requires a stopband width of 1 GHz. Next, the implementation of the wide stopband of the open-loop resonator will be analyzed. The following and 29 presents the characteristic equation for the relationship between the fundamental resonance frequency and the dispersive resonance frequency:


$$\begin{array}{c} \theta_{D1}=2\tan^{-1}\frac{1}{\pi f_{D1}Z_{R}C_{R}} \end{array}$$ (28)

$$\begin{array}{c} \theta_{D2}=2\pi -2\tan^{-1}(\pi f_{D2}Z_{R}C_{R}) \end{array}$$ (29)

Here, *f *D1 is the fundamental resonance frequency of *f *D, *f *D2 is the dispersive resonance frequency of *f *D, ZR is the total impedance of the open-loop resonator, and CR is the capacitive coupling generated by the coupling distances S1 and S2. and 29 are both monotonically decreasing. When CR increases, that is, when the coupling distances S1 and S2 are shortened, *f *D1 and *f *D2 both increase if the electrical length remains unchanged. These equations illustrate that the response of the actual resonator is not only related to the device’s dimensional parameters but also to the impedance values ZR and CR introduced by the material. This paper focuses more on how the open-loop resonator achieves a wide stopband, thus observing the changes in the ratio of *f *D1/*f *D2 in .


θD2θD1= arctan ⁡ 2θD1 arctan ⁡ 22π−θD2 (30)

**Fig 20:**
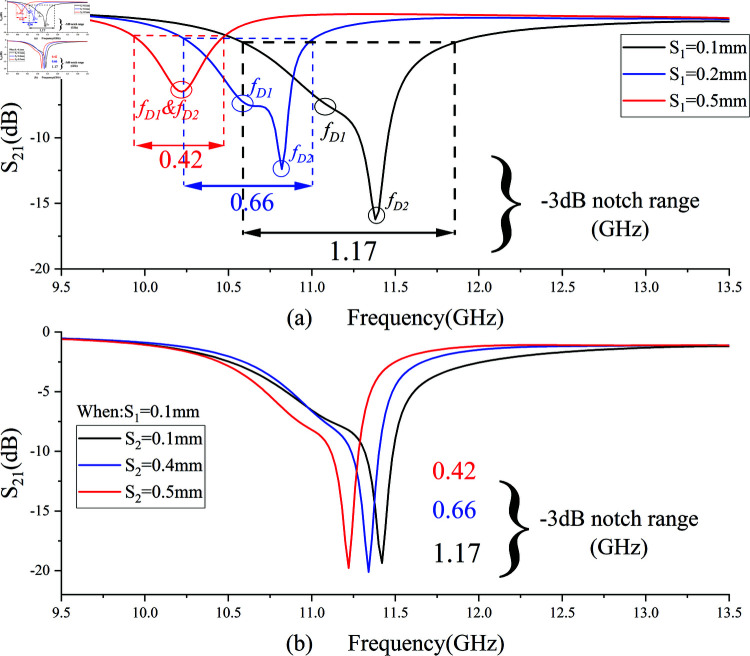
Effect of varying coupling distances S_1_ and S_2_ on the wide stopband phenomenon.

When the electrical lengths θ_1_ and θ_2_ are shortened, the center frequencies *f *D1 and *f *D2 shift to higher frequencies. As long as the center frequency *f *D2 shifts more, the *f *D1/*f *D2 ratio increases, making the wide stopband phenomenon more likely. Fig 20 presents experimental results to verify the phenomena described by and 30, aiding in better understanding and designing the fourth notch frequency *f *D.

In Fig 20(a), it is clearly observed that when S1 is 0.5 mm, the stopband resonance frequency has only one negative peak, with no dispersion occurring. As the coupling distance S1 decreases to 0.2 mm, a local negative peak *f *D1 and a larger negative peak *f *D2 appear. With further reduction of S1, *f *D1 and *f *D2 show a clear separation trend. As *f *D1 and *f *D2 increase, the –3 dB notch range achieved by the open-loop resonator also significantly increases, reaching a notch band of 1.17 GHz. Observing Fig 20(b), fixing S1 at 0.1 mm and adjusting S2 also shows that the –3 dB notch range gradually increases but the selectivity slightly decreases. However, considering that when S2 is 0.1 mm, the coupling distance between the open-loop resonator and CPW ground is too small, which significantly increases the high-frequency passband loss, S2 is chosen to be 0.2 mm.

**Fig 21:**
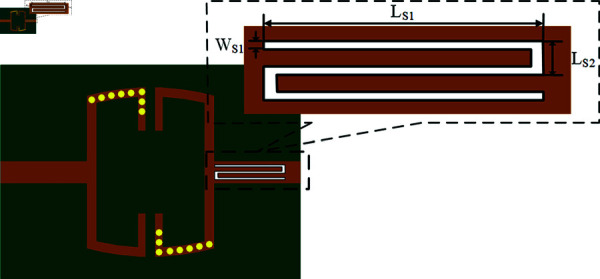
The inverted S-shaped EBG.

Next, we will construct the fifth notch structure, *f *E, to eliminate the signal frequency band used by the Starlink megaconstellation. Currently, SpaceX has obtained FCC [25] authorization for the Ku-band uplink frequency of 12.75–13.25 GHz (Earth-to-space). Since the CPW slot line area is fully occupied, an Electromagnetic Bandgap (EBG) structure with frequency selectivity can be embedded at the output end of the coupled microstrip transmission line to achieve the five notch waves filter. The EBG is characterized by enhanced stopband strength and a small size [31]. Compared to other centrally symmetrical notch structures, the fifth notch filter fE uses an embedded inverted S-shaped EBG. The EBG is placed only at the output end to protect the original coupling path from being disrupted, thereby reducing the impact on passband smoothness. Fig 21 shows the inverted S-shaped EBG.

The left side of Fig 21 shows the inverted S-shaped EBG structure and its placement area. The stopband control parameters include the slot line width WS1, and the total length is determined by LS1 and LS2. The right side requires sufficient space at the output end for soldering.

**Fig 22:**
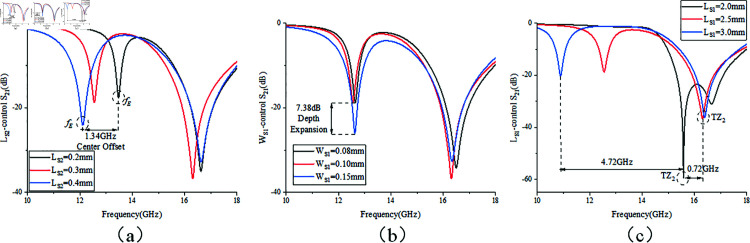
Effect of three control parameters of the inverted S-shaped EBG. (a) LS2. (b) WS1. (c) LS1.

Changing the three size parameters WS1, LS1, and LS2 of the inverted S-shaped EBG in Fig 21 will affect its performance, as shown in Fig 22. In Fig 22(a), increasing LS2 leads to a significant expansion in the frequency range of the notch band, with a slight improvement in selectivity. The center frequency *f *E of the fifth notch band shifts slightly towards the lower frequency. In Fig 22(b), an increase in WS1 results in a modest expansion of the notch band frequency range and a slight increase in selectivity. When WS1 is increased to 0.15 mm, the notch depth significantly increases, achieving an enhanced suppression depth of up to –7.38 dB, but the high-frequency passband loss also increases noticeably. In Fig 22(c), increasing LS1 does not affect the frequency range and selectivity of the notch band, but it causes the center frequency *f *E to shift significantly towards the lower frequency. Notably, when LS2 is set to 2.5 mm, the stopband center frequency fE coincides with the original transmission zero TZ2, resulting in a rapid roll-off reduction of 0.72 GHz and stronger selectivity at 15.58 GHz, with –30 dB stopband suppression improving from 0.86 to 0.93. Based on the results of the three parameters in Fig 22, the key parameters for tuning the center frequency *f *E of the fifth notch band are LS1 and LS2, while WS1 and LS2 are critical for adjusting the notch band frequency range and selectivity. To achieve a notch band with a frequency range of 12.75 GHz to 13.25 GHz and –10 dB selectivity, the corresponding parameter values are LS1 = 2.6 mm, LS2 = 0.3 mm, and WS1 = 0.1 mm.

**Fig 23:**
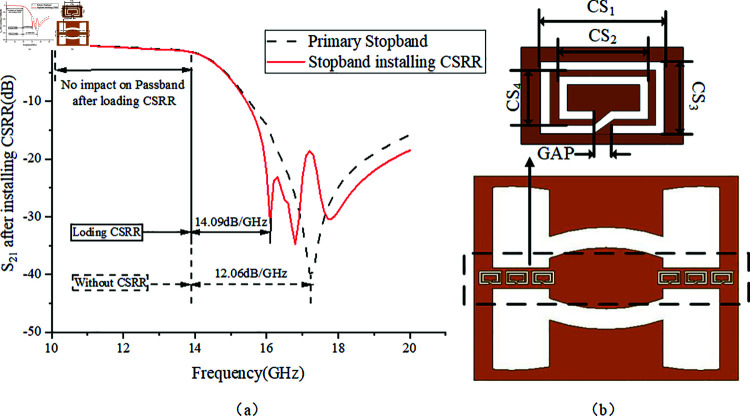
Series complementary resonant rings. (a) Mounting Effect. (b) Embedded Position.

After completing the analysis and design of the coupling path for multimode passband and multiple stopband notch structures, the final step is to enhance the –30 dB stopband rejection by adding a series complementary resonant ring structure in the stopband where TZ2 is located, as shown in Fig 23.

Fig 23 illustrates the use of a series of Complementary Split Ring Resonators (CSRR) as a defected ground structure to enhance the selectivity of the stopband. It can be observed that embedding the series CSRR not only does not increase the size of the filter but also has no impact on the –3 dB passband insertion loss. Additionally, it shortens the –30 dB stopband range, increasing the stopband roll-off speed from 12.06 GHz to 14.09 GHz.The parameters for the series complementary resonant rings that achieve optimal stopband performance are: CS1 = 1.2 mm, CS2 = 0.8 mm, CS3 = 0.7 mm, CS4 = 0.4 mm, GAP = 0.2 mm.

## Current distribution

**Fig 24:**
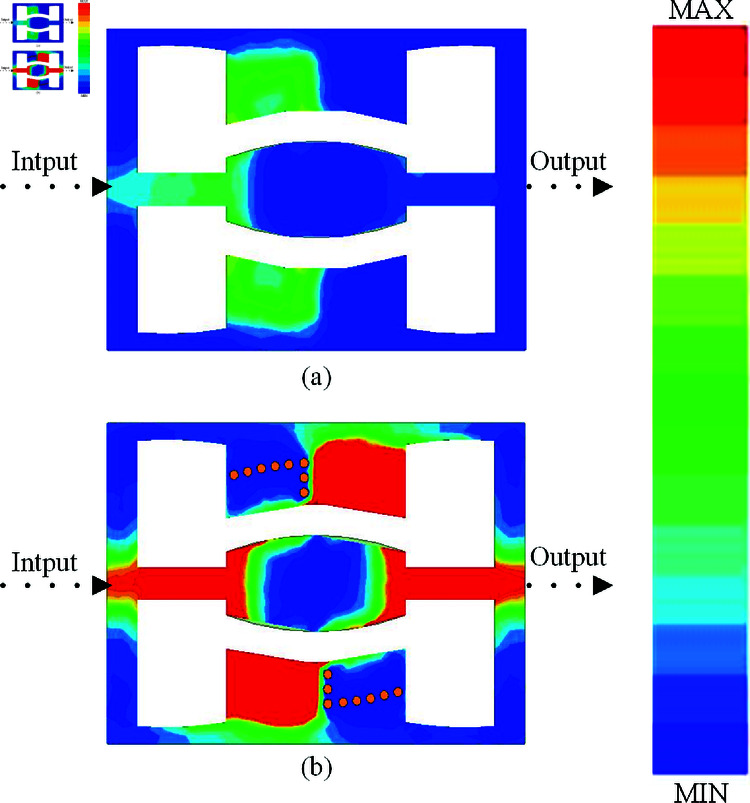
Current Distribution of the Prototype Filter at 2.4 GHz.

Upon completing the design processes, a current distribution analysis was conducted to validate the design’s effectiveness. This analysis is presented in three sections, corresponding to the design steps:

1. Comparative current distribution of the prototype filter with and without the SVA structure, as shown in Fig 24.
2. Current distribution analysis across the six passbands, illustrated in Fig 2.
3. Current distribution analysis of the five notch bands, depicted in Fig 3.

As shown in Fig 24(a), the current distribution for the CPW circuit without the SVA structure does not reach the output end at the operating frequency of 2.4 GHz, with the maximum current phase not fully propagating. However, in Fig 24(b), with the SVA structure integrated, the CPW circuit achieves not only extended current reach to the output but also an increase in current intensity, demonstrating the advantage of the SVA-ML-CPW structure in enhancing bandwidth without additional size.

**Fig 25:**
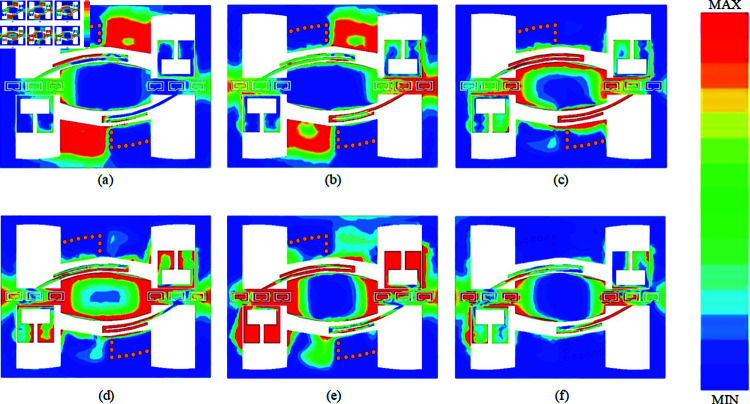
Electric Field Spectrum of the Proposed Filter at the Center Frequencies of the Six Passbands, a) 3.08 GHz, (b) 4.65 GHz, (c) 5.98 GHz, (d) 8.77 GHz, (e) 11.88 GHz, (f) 13.74 GHz.

Fig 25 presents the electric field spectra of the proposed hexa-passband, five-notch-band filter at each passband center frequency: (a) 3.08 GHz, (b) 4.65 GHz, (c) 5.98 GHz, (d) 8.77 GHz, (e) 11.88 GHz, and (f) 13.74 GHz. As observed, the filter operates with two distinct signal paths. Specifically, at the working frequencies in Fig 25(a) and Fig 25(b), the SVA-ML-CPW structure serves as the signal path for the passbands, whereas for Fig 25(c) to Fig 25(f), the ML-CPW structure supports the signal path for the respective passbands.

**Fig 26:**
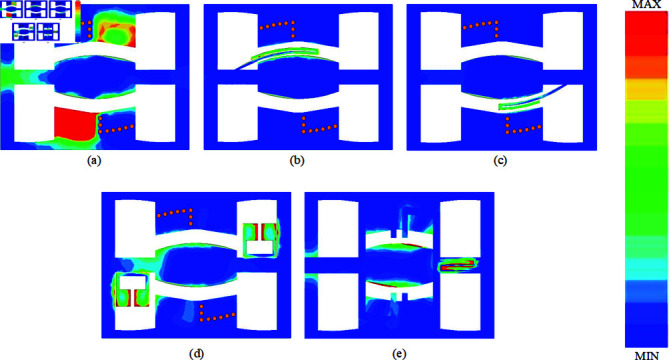
Electric Field Spectrum of the Proposed Filter at the Center Frequencies of the Five Notch Bands, (a) 3.96 GHz, (b) 5.45 GHz, (c) 6.86 GHz, (d) 11.28 GHz, (e) 12.68 GHz.

Fig 26 illustrates the electric field spectra of the five notch bands for the proposed hexa-passband, five-notch-band filter at the following center frequencies: (a) 3.96 GHz, (b) 5.45 GHz, (c) 6.86 GHz, (d) 11.28 GHz, and (e) 12.68 GHz. Notably, in each notch band center frequency, the electric field spectrum shows that, upon integrating the notch structure, energy entering from the left input fails to propagate through to the output on the right. Resonance occurs due to the corresponding notch structures, effectively achieving notch band formation and signal suppression.

## Testing and verification

**Fig 27:**
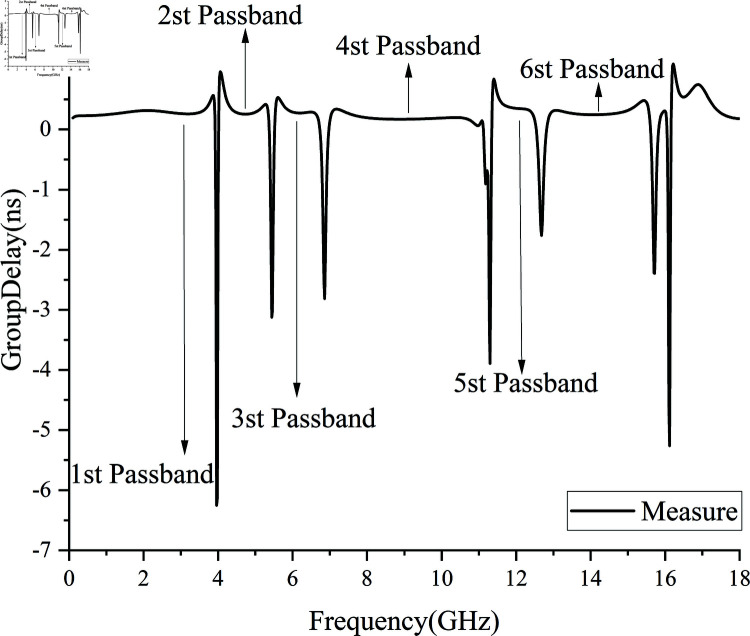
Group delay.

**Fig 28:**
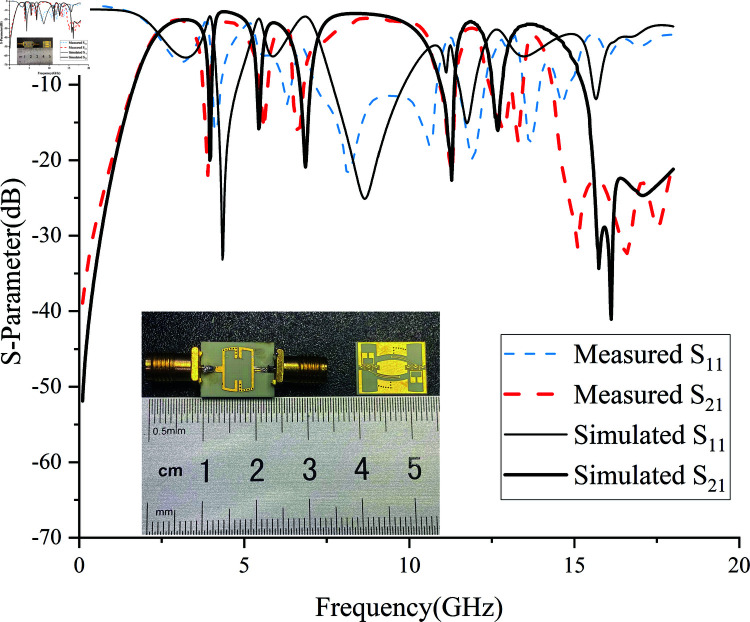
Series complementary resonant rings. (a) Mounting Effect. (b) Embedded Position.

Based on the new coupling path, a wideband S/C/X/KU band filter integrating five notch structures to suppress commercial communication signals was designed, simulated, and optimized using ANSYS Electronics 2022.2 HFSS. The fabricated filter was tested using a Ceyear 3674H vector network analyzer, The group delay results are shown in Fig 27, and the S-parameter test results are presented in Fig 28. The filter was fabricated using high-frequency PCB technology, with a Rogers 4350C dielectric substrate, relative permittivity of 3.66, loss tangent of 0.004, and a standard thickness of 0.508 mm (20 mil). The physical dimensions of the five notch filter are 10.8×14 *mm*^2^, with a relative waveguide size of 0.38 *× 0.29λ*^2^ (where *λ* corresponds to a center frequency of 8.5 GHz and a waveguide wavelength of 36.58 mm).

Fig 27 shows that the maximum group delay across the five passbands does not exceed 0.33 ns, with specific values as follows: the first passband is 0.25 ns, the second passband is 0.25 ns, the third passband is 0.27 ns, the fourth passband is 0.33 ns, and the fifth passband is 0.25 ns. The low group delay reduces phase distortion, resulting in enhanced stability of the filter.

Fig 28 shows that the measured –3 dB passband range of the filter is from 2.4 GHz to 14 GHz, with a total bandwidth of 11.6GHz. The return loss is greater than –11.5 dB, and the relative bandwidth reaches 141%. With the integration of five notch structures, the center frequency of the passband is at 8.325 GHz, and the insertion loss is only –1.43 dB. The –30 dB stopband rejection is 0.92. The measured center frequencies of the five notches, *f *A, *f *B, *f *C, *f *D, and *f *E, are 3.96 GHz, 5.45 GHz, 6.86 GHz, 11.28 GHz, and 12.68 GHz, respectively. The suppression depths are –20.01 dB, –15.84 dB, –20.90 dB, –22.65 dB, and –16.04 dB, respectively. The results indicate a good agreement between the theoretical, simulated, and measured values.

The measured S-parameters and simulated S-parameters showed two different comparative results beyond 12.68 GHz. The first discrepancy is that the measured high-frequency passband is 0.26 GHz narrower than the simulated passband but still maintains high selectivity. This is due to the insertion loss caused by manually soldering the SMA connectors and connecting coaxial cables, as well as the manufacturing precision errors of the coupling distances S1 and S2 of the CSRR. The second discrepancy is that while the fifth notch *f *E functions correctly, two notch peaks appear. This is because the inverted S-shaped EBG is very close to the manual soldering points, and the solder joints leave air gaps with the coaxial cables, resulting in two resonance centers. The results indicate that the measured filter demonstrates excellent wideband performance and multiple notch capabilities while maintaining low loss in the passband and high selectivity. There is good consistency between theoretical, simulated, and measured values.

**Table 1:** Comparison of the parameters of this filter and reference filter.

Ref.	Main structures	P.B.(GHz)	FBW/%	Notches	Notch bands (GHz)	I.F.(dB)	–30 dB S.F.	Size($\lambda_{g}\times \lambda_{g}$)
Wideband filter without notch or FBW less than 100%								
[31]	SIW	4.9–8.7	57.6	0	NA	–0.25	NA	0.59*0.45
[32]	CC-SIR	4.3–7.0	58.5	0	NA	–1.1	0.8	0.77*0.70
[33]	MPCB	10.215–15.375	40.33	0	NA	–0.58	NA	0.89*0.85
[34]	SIW+SIR	3.3–10.57	104.3	0	NA	–1.4	NA	0.25*0.16
[35]	HPF+LPF	2.92–10.95	107	0	NA	–0.49	0.78	0.28*0.25
Notch wideband filter with FBW exceeding 100%								
[36]	ML-CPW	3.25–10.73	103	3	5.6/6.42/ 8.03	–1.6	0.92	1.04*0.66
[37]	TS-MMR	2.8–10.6	116	3	3.2/5.0/ 8.2	–0.8(Sim)	NA	0.76*0.27
[38]	TS-MMR	3.1–10.4	108	4	5.3/5.9/6.4/7.4	NA	NA	0.61*0.34
[39]	SIR	2.4–10.5	125	1	5.1	–3	0.88	0.77*0.68
[40]	ML-CPW	2.9–10.5	113	2	4.55/8.07	–1.15	0.89	0.44*0.33
[41]	ML-CPW	3.76–11.29	110	2	9.28/10.48	–1.46	0.93	0.20*0.20
[42]	TS-SIR	2.7–12.1	127	3	5.1/6.0/8.0	–1	NA	0.85*0.37
This work	SVA-ML-CPW	2.4–14.3	141	5	3.96/5.45/ 6.86/11.28/12.68	–1.43	0.92	0.38*0.29
Multi-band wideband filter: Penta-band filter and Hexa-band filter								
Ref.	Main structures	C.F. (GHz)	FBW/% age	P.B.s	Maximum I.F. (dB)	Maximum accuracy of band (GHz)	Group delay (ns)	Size($\lambda_{g}\times \lambda_{g}$)
[39]	TS-MMR	4.2/5.75/6.13/6.77/8.74	50.47/7.82/4.08/7.25/33.41	5	0.88/0.7/0.63/0.37/1.87	0.1	NA	0.61*0.34
[43]	SCSRR	1.8/2.4/3.5/4.9/5.8	13.4/12.6/15.8/9.4/9.85	5	1.1/0.95/1.65/1.5/1.65	0.1	NA	0.17*0.10
[44]	SIR	1.8/2.5/3.3/3.8/4.5	11/15/5/5/5	5	0.7/0.28/0.8/0.6/0.9	0.1	NA	0.32*0.19
[45]	TS-MMR	1.8/3.5/4.5/5.5/6.8	4.8/12/1.5/4.5/0.59	5	0.7/0.28/0.8/0.6/0.9	0.1	NA	0.18*0.15
[46]	SIR	1.5/2.5/3.5/4.5/5.8	4.5/4.5/3.6/4.5/2.7	5	1.5/1.8/0.9/1.2/2.5	0.1	NA	0.24*0.17
[47]	SL-SIR	1.06/3.24/5.36/8.55/10.78	25.59/22.22/19.48/16.03/17.17	5	0.35/0.37/0.17/0.19/0.20	0.01	1.25/0.65/0.38/0.34/0.68	0.23*0.14
[48]	TS-MMR	2.4/5.0/7.0/8.8/10.5/12.9	NA	6	0.1/0.2/0.5/0.5/1.0/2.0	0.1	NA	0.89*0.74
[49]	SIR	1.7/3.48/5.08/6.5/8.66/10.18	19.4/9.2/30/6.7/10.2/13	6	0.66/1.33/1.19/1.38/1.3/0.93	0.1	NA	0.20*0.17
[50]	Semilumped-R	0.8/1.1/1.4/1.7/2/2.3	2.6/2.3/2/1.8/1.6/1.3	6	2.13/2.3/2.26/2.18/2.08/2.76	0.01	NA	0.74*0.06
This work	SVA-ML-CPW	3.08/4.65/5.98/8.77/ 11.88/13.74	42.9/24.3/10.7/33.5/5.4/8.0	6	1.37/0.53/1.43/0.55/1.64/1.9	0.001	0.25/0.25/0.27/0.17/ 0.33/0.25	0.38*0.29

Ref.: Reference; P.B.: Pass band; FBW: fractional bandwidth; NA: no available; Sim: Simulation data; *λ*_g_: Relative waveguide wavelength at the bandwidth center frequency; I.F.: insertion loss; S.F.: Sharpness factor; C.F.: Center frequencies.

Table 1 presents a comparison of the proposed filter with recent microstrip wideband filters [19,32–42]. The second column of the table indicates that the proposed filter utilizes a different filter structure, employing a SVA-ML-CPW configuration to achieve FBW of up to 141%. Compared to filters reported in references [32–36], although the insertion loss of the proposed filter is slightly increased, its FBW is nearly 1.5 times to almost 3 times larger. Furthermore, despite integrating notch structures, the filter’s dimensions remain advantageous compared to those without notch structures. In comparison with references [36–42], the proposed filter achieves the highest number of five notch frequencies, maintains insertion loss at a normal level, and has a notable advantage in the –30 dB stopband rejection. Additionally, the filter integrates these features with a relatively compact overall size. While filters in references [36–42] typically achieve either narrowband or wideband notch rejection, the proposed filter simultaneously provides both narrowband and wideband rejection. Furthermore, the study analyzes the higher-order mode phenomena in the notch rejection structures and offers methods to mitigate their impact on the passband. Overall, the primary advantage of the proposed filter is its ability to realize up to five notch frequencies with a compact size, while maintaining high selectivity and only a modest increase in insertion loss.

The third section of the table compares recent Penta-band and Hexa-band filters[39,43–50]. It can be observed that, although the proposed filter does not particularly stand out in terms of insertion loss and size, it demonstrates a significant advantage in frequency range accuracy and passband coverage. Specifically, the proposed filter achieves a frequency range accuracy of 0.001 GHz, whereas the frequency selection precision of references [39,43–46,48] only reaches 0.1 GHz, and references [47,49,50] reach 0.01 GHz. Additionally, the passbands of references [43–46,50] are primarily applied in the S/C bands, while references [39,47–49] are applied in the S/C/X bands. In contrast, the passband of the proposed filter covers the S/C/X/Ku bands, providing a broader selection of passband options. Furthermore, the fractional bandwidth (FBW) of each passband in the proposed filter is significantly wider than that in references [39,43–50].

The proposed filter also includes group delay analysis, validating its stability, similar to reference [47]. References [44,46,47], which employ a stepped impedance resonator (SIR) structure, generate all notch bands using transmission zeros. Consequently, the range and center frequency of each notch band are interdependent, causing the center frequencies of each notch band to shift in synchrony. By contrast, the proposed filter benefits from independently designed notch structures, enabling independent control of the range and center frequency of each notch band without mutual interference.

Further analysis of references [47,50] shows that while these designs achieve relatively high frequency range precision, they have certain limitations. Reference [47] adjusts the physical length of short-circuited stubs to control multiple passband ranges. However, to maintain the strength of the lowest frequency transmission zero, phase stability is compromised, resulting in a maximum group delay of 1.25 ns. In contrast, the maximum group delay of the proposed filter is only 0.33 ns, offering superior stability. Reference [50] achieves six signal paths using six pairs of semi-lumped resonators, allowing precise frequency control. However, extensive coupling causes an insertion loss exceeding 2.3 dB.

In summary, the proposed single-layer microstrip filter provides superior frequency range accuracy, broader spectrum coverage, a higher number of up to six passbands, and notably enhanced stability compared to other Penta-band and Hexa-band filters.

## Results

This manuscript introduces a bandpass filter for the S/C/X/KU band based on a novel SVA-ML-CPW structure with new coupling paths. The filter design involves analyzing and designing five passband modes and using weak coupling modes to achieve five well-distributed passband modes. Additionally, a miniaturized dual-band stub structure is proposed, which offers excellent narrowband rejection performance and a flexible adjustable frequency range in the C-band. The manuscript also presents an analysis and solution for high-order harmonic interference associated with this structure. The five notch suppression effect for commercial communication signals in the S/C/X/KU band is achieved by integrating the dual-band stub structure, transmission zero TZ1, open-loop resonator, and inverted S-shaped EBG structure. For high selectivity at the passband edges, transmission zero TZ2 and a series of CSRR are used. Overall, the bandpass filter is designed to be compact with outstanding multiple notch performance and miniaturization advantages.

## References

[1] Rahayu Y, Abd Rahman T, Ngah R, Hall PS. Ultra wideband technology and its applications. Proceedings of the 2008 5th IFIP International Conference on Wireless and Optical Communications Networks (WOCN’08). IEEE; 2008. p. 1–5.

[2] Yoza-Mitsuishi N, Mathys P. Considerations for spectrum sharing between RLANs and incumbents in the 13 GHz band. 2021 IEEE 94th Vehicular Technology Conference (VTC2021-Fall). IEEE; 2021. p. 1–6. doi: 10.1109/vtc2021-fall52928.2021.9625584

[3] Radio Communication Sector. Report itu-r f. 2323-2. 2023.

[4] Abubakar HS, Zhao Z, Munir ME, Tareen WUK, Wang B, Kiani SH, et al. Enhanced smartphone connectivity: dual-band MIMO antenna with high isolation and low ECC. Phys Scr 2024;99(6):065524. doi: 10.1088/1402-4896/ad4424

[5] Nan L, Chunxia G, Dapeng W. Considerations on 6 GHz spectrum for 5G-advanced and 6G. IEEE Comm Stand Mag. 2021;5(3):5–7. doi: 10.1109/mcomstd.2021.9579392

[6] Zhu L, Sun S, Li R. Microwave bandpass filters for wideband communications. Wiley; 2011.

[7] Hunter I. Theory and design of microwave filters. Number 48. Iet, 2001.

[8] Aiello R, Rogerson GD. Ultra-wideband wireless systems. IEEE Microw Magaz. 2003;4(2):36–47.

[9] Packiaraj D, Vinoy K, Ramesh M, Kalghatgi A. Miniaturized ultra wide band filter with extended stop band. Microw Optic Technol Lett. 2013;55(4):703–5.

[10] Zheng X, Pan Y, Jiang T. UWB bandpass filter with dual notched bands using T-shaped resonator and L-shaped defected microstrip structure. Micromachines (Basel) 2018;9(6):280. doi: 10.3390/mi9060280 30424213 PMC6187688

[11] Chen D, Bu H, Zhu L, Cheng C. A differential-mode wideband bandpass filter on slotline multi-mode resonator with controllable bandwidth. IEEE Microw Wirel Compon Lett. 2014;25(1):28–30.

[12] Snyder RV, Mortazawi A, Hunter I, Bastioli S, Macchiarella G, Wu K. Present and future trends in filters and multiplexers. IEEE Trans Microw Theory Techniq. 2015;63(10):3324–60.

[13] Parthasarathy R, Arumugam C, Venkatesan RP, Ponnusamy M. Design of compact uwb filter using parallel-coupled line and circular open-circuited stubs. IETE J Res. 2022;68(6):4665–72.

[14] Gao X, Feng W, Che W. High-selectivity wideband balanced filters using coupled lines with open/shorted stubs. IEEE Microw Wirel Components Lett. 2017;27(3):260–2.

[15] Guo X, Zhu L, Wu W. Strip-loaded slotline resonators for differential wideband bandpass filters with intrinsic common-mode rejection. IEEE Trans Microw Theory Techniq. 2016;64(2):450–8.

[16] Munir ME, Nasralla MM, Esmail MA. Four port tri-circular ring mimo antenna with wide-band characteristics for future 5g and mmwave applications. Heliyon, 10(8), 2024. 10.1016/j.heliyon.2024.e28714 PMC11024564 38638997

[17] Sethi W, Kiani SH, Munir ME, Sehrai DA, Awan D. Pattern diversity based four-element dual-band mimo patch antenna for 5g mmwave communication networks. J Infrared Millimete Terahertz Waves. 2024;45(5):521–37.

[18] Mehr E, Kiani SH, Savci HS, Sehrai DA, Muhammad F, Ali A, et al. mmwave polarization diversity wideband multiple-input/multiple-output antenna system with symmetrical geometry for future compact devices. Symmetry. 2023:15(9);1641.

[19] Ghazali MS, Pal S, Abu Nasar. Multiple passband transmission zeros embedded compact uwb filter based on microstrip/cpw transition. AEU-Int J Electron Commun. 2021;129:153549.

[20] Ma K, Ma J-G, Yeo KS, Do MA. A compact size coupling controllable filter with separate electric and magnetic coupling paths. IEEE Trans Microw Theory Techniq. 2006;54(3):1113–9.

[21] Srinivasa Rao R. Microwave engineering. PHI Learning Pvt. Ltd., 2015.

[22] Ghazali AN, Sazid M, Pal S. A compact broadside coupled dual notched band UWB-BPF with extended stopband. AEU – Int J Electron Commun. 2017;82:502–7. doi: 10.1016/j.aeue.2017.10.021

[23] Sehrai DA, Munir ME, Kiani SH, Shoaib N, Algarni AD, Elmannai H, et al. A high gain array based millimeter wave mimo antenna with improved isolation and decorrelated fields. IEEE Access. 2024.

[24] Ghosh M. Sharing in the 12 GHz Band. IEEE Wireless Commun. 2023;30(3):10–1. doi: 10.1109/mwc.2023.10183719

[25] Mozaffariahrar E, Theoleyre F, Menth M. A survey of Wi-Fi 6: technologies, advances, and challenges. Future Internet 2022;14(10):293. doi: 10.3390/fi14100293

[26] Axelsson M. Design of a satellite constellation intended for use with a small user terminal. 2022.

[27] Kiani SH, Munir ME, Savci HS, Rimli H, Alabdulkreem E, Elmannai H, et al. Dual-polarized wideband 5g n77 band slotted mimo antenna system for next-generation smartphones. IEEE Access. 2024.

[28] Song K, Xue Q. Compact ultra-wideband (UWB) bandpass filters with multiple notched bands. IEEE Microw Wireless Compon Lett. 2010;20(8):447–9. doi: 10.1109/lmwc.2010.2050303

[29] Hong J-S, Lancaster MJ. Theory and experiment of novel microstrip slow-wave open-loop resonator filters. IEEE Trans Microw Theory Techniq. 1997;45(12):2358–65.

[30] Seymour BC. Direct-coupled-resonator filters. Proc IRE. 1957;45(2):187–96.

[31] Huang SY, Lee YH. Compact u-shaped dual planar ebg microstrip low-pass filter. IEEE Trans Microw Theory Techniq. 2005;53(12):3799–805.

[32] Liu F, Yang L, Cai B, Wu L, Cheng Y, Chen F, et al. Compact and miniaturized wideband bandpass filter based on substrate integrated waveguide and microstrip line. J Appl Phys. 2024;135(24).

[33] Abdul Rehman M, Khalid S, Mushtaq B, Idrees M. Design of a novel compact highly selective wideband bandstop RF filter using dual path lossy resonator for next generation applications. PLoS One 2022;17(10):e0273514. doi: 10.1371/journal.pone.0273514 36315491 PMC9621440

[34] Li C, Ma Z-H, Chen J-X, Wang M-N, Huang J-M. Design of a compact ultra-wideband microstrip bandpass filter. Electronics. 2023:12(7);1728.

[35] Hussain Bohra H, Ghosh A, Bhaskar A. Design and analysis of spurious harmonics suppressed microstrip ultrawide band filter using modified defected ground structure techniques. Wirel Personal Commun. 2021;121(1):361–80.

[36] Jamsai M, Angkawisittpan N, Nuan-On A. Design of a compact ultra-wideband bandpass filter using inductively compensated parallel-coupled lines. Electronics. 2021;10(21):2575

[37] Sazid M, Raghava N. Planar uwb-bandpass filter with multiple passband transmission zeros. AEU-Int J Electron Commun. 2021;134:153711.

[38] Basit A, Khattak MI, et al. Design and analysis of a microstrip planar uwb bandpass filter with triple notch bands for wimax, wlan, and x-band satellite communication systems. Prog Electromagnet Res M. 2020;93:155–64.

[39] Liu F, Qun M. A new compact uwb bandpass filter with quad notched characteristics. Prog Electromagnet Res Lett. 2020;88:83–8.

[40] Liu L-Q, Lai H-S, Hu H-M, Chen J-J, Weng M-H, Yang R-Y. A simple method to design a UWB filter with a notched band using short-circuit step impedance stubs. Electronics 2022;11(7):1124. doi: 10.3390/electronics11071124

[41] Gao M, Zhang X, Chen X, Nan J. Design of double-notch UWB filter with upper stopband characteristics based on ACPW-DGS. PLoS One 2023;18(2):e0282060. doi: 10.1371/journal.pone.0282060 36812186 PMC9946202

[42] Basit A, Khattak MI, Zubir F, Shah SW. Miniaturized ultra-wideband filter with independently controlled notch bands for 5.1/6/8 GHz wireless applications. PLoS One. 2022;17(6):e0268886. doi: 10.1371/journal.pone.0268886 35679270 PMC9182340

[43] Ramanujam P, Ramanujam K, Ponnusamy M. A novel asymmetrical interdigital coupled line-based penta-band bandpass filter design with enhanced selectivity employing square complementary split ring resonator. Int J RF Mic Comp-Aid Eng 2021;31(12):e22888. doi: 10.1002/mmce.22888

[44] Chen Y-W, Wu H-W, Chen Y-W, Liu R, Ye H, Liu S-K. Design of new compact multi-layer quint-band bandpass filter. IEEE Access. 2021;9:139438–45. doi: 10.1109/access.2021.3116807

[45] Wang L, Yang S, Zhang L, Li B. Design of a compact quint-band bandpass filter using a symmetric dual-mode *λ*/4 resonator and a pair of inverted F-shaped resonators. J Electromagnet Waves Appl. 2022;36(16):2289–304. doi: 10.1080/09205071.2022.2073475

[46] Hsu K-W, Hung W-C, Tu W-H. Compact quint-band microstrip bandpass filter using double-layered substrate. In: 2013 IEEE MTT-S International Microwave Symposium Digest (MTT). IEEE; 2013. p. 1–4. doi: 10.1109/mwsym.2013.6697353

[47] Mushtaq B, Khalid S, Rehman MA. Design of a compact novel stub loaded pentaband bandpass filter for next generation wireless RF front ends. IEEE Access. 2022;10:109919–24. doi: 10.1109/access.2022.3214313

[48] Gaswin KG, Sreeja MS. Design of hexa-band microwave bandpass filter using modified t shaped multimode resonator. In: AIP Conference Proceedings. AIP Publishing; 2023. p. 2690.

[49] Li X, Zhang Y, Tian Y, Yang Y, Fan Y. Quad-and sext-band bandpass filter based on multimode resonator utilizing sir s-loaded tapered-line. Microw Opt Technol Lett. 2018;60(3):650–4.

[50] Tu W-H, Hsu K-W. Design of sext-band bandpass filter and sextaplexer using semilumped resonators for system in a package. IEEE Trans Components Packag Manufact Technol. 2015;5(2):265–73.

